# ATL9, a RING Zinc Finger Protein with E3 Ubiquitin Ligase Activity Implicated in Chitin- and NADPH Oxidase-Mediated Defense Responses

**DOI:** 10.1371/journal.pone.0014426

**Published:** 2010-12-23

**Authors:** Marta Berrocal-Lobo, Sophia Stone, Xin Yang, Jay Antico, Judy Callis, Katrina M. Ramonell, Shauna Somerville

**Affiliations:** 1 Department of Plant Biology, Carnegie Institution, Stanford, California, United States of America; 2 Department of Biological Sciences, University of Alabama, Tuscaloosa, Alabama, United States of America; 3 Section of Molecular and Cellular Biology, University of California Davis, Davis, California, United States of America; University of California, United States of America

## Abstract

Pathogen associated molecular patterns (PAMPs) are signals detected by plants that activate basal defenses. One of these PAMPs is chitin, a carbohydrate present in the cell walls of fungi and in insect exoskeletons. Previous work has shown that chitin treatment of *Arabidopsis thaliana* induced defense-related genes in the absence of a pathogen and that the response was independent of the salicylic acid (SA), jasmonic acid (JA) and ethylene (ET) signaling pathways. One of these genes is *ATL9* ( = *ATL2G*), which encodes a RING zinc-finger like protein. In the current work we demonstrate that ATL9 has E3 ubiquitin ligase activity and is localized to the endoplasmic reticulum. The expression pattern of *ATL9* is positively correlated with basal defense responses against *Golovinomyces cichoracearum*, a biotrophic fungal pathogen. The basal levels of expression and the induction of *ATL9* by chitin, in wild type plants, depends on the activity of NADPH oxidases suggesting that chitin-mediated defense response is NADPH oxidase dependent. Although *ATL9* expression is not induced by treatment with known defense hormones (SA, JA or ET), full expression in response to chitin is compromised slightly in mutants where ET- or SA-dependent signaling is suppressed. Microarray analysis of the *atl9* mutant revealed candidate genes that appear to act downstream of *ATL9* in chitin-mediated defenses. These results hint at the complexity of chitin-mediated signaling and the potential interplay between elicitor-mediated signaling, signaling via known defense pathways and the oxidative burst.

## Introduction

Plants defend against pathogens using an innate system of defense that has both constitutive and inducible components. Constitutive defense responses are independent of the physical presence of a pathogen and are typically chemical and physical barriers that protect the plant from pathogen invasion [1]. Inducible plant defenses depend on pathogen recognition and fall into two major classes; specific gene-for-gene interactions and more general Pathogen or Microbe-Associated Molecular Pattern (PAMP or MAMP)-associated responses. In gene-for-gene interactions, a plant resistance (*R*) gene recognizes and interacts with a specific race(s) of pathogen that expresses a corresponding avirulence (*Avr*) gene [2]. In the absence of gene-for-gene interactions, general elicitors or PAMPs, [3] are recognized by the host and activate broad-spectrum defense responses. Common PAMPs such as the oligosaccharide chitin (β-1, 4 linked *N-*acetylglucosamine) and the bacterial proteins flagellin and elongation factor Tu (EF-Tu) are known to activate strong defense responses [4], [5], [6], [7], [8]. Several receptors associated with these PAMPs have been characterized including: the FLS2 receptor that recognizes flagellin [9]; the EFR receptor, which perceives the first 18 amino acids of bacterial elongation factor Tu (EF-Tu) [7], chitin oligosaccharide elicitor binding protein (CeBiP) [10], a transmembrane protein with two extracellular Lysine motifs (LysM) that is involved in chitin recognition and the LysM-RLK CERK1 that is required for chitin-initiated responses and downstream signalling [10], [11].

While our knowledge of how plants perceive pathogens and activate associated defense signaling pathways is increasing rapidly, less is known about how these processes are regulated during the infection. A predominant theme that is emerging is that of ubiquitination as a means of targeting components of defense signaling pathways for degradation to curtail the plant immune response [12], [13]. Studies characterizing the roles of several ubiquitin E3 ligases in defense have begun to provide clues about the regulation of pathogen-induced signaling [12], [14]. For instance the rice resistance (R) protein Xa21 has been shown to interact with an E3 ubiquitin ligase XB3 [15]. Interaction between XB3 and Xa21 is required for the accumulation of the XA21 protein and is necessary for Xa21-mediated resistance to *Xanthomonas oryzae pv. oryza* in rice [15], [16]. A RING-finger type protein from pepper CaRFP1 was shown to physically interact with PR-1 (pathogenesis related-1) protein in leaves of plants after infection with both bacterial and fungal pathogens [17]. Over-expression of *CaRFP1* in transgenic Arabidopsis conferred disease susceptibility to *Pseudomonas syringae* pv. *tomato* and reduced *PR-2* and *PR-5* expression suggesting that CaRFP1 is an E3 ligase that targets PR proteins [17].

E3 ligases also appear to play a prominent role in elicitor-mediated defense responses. In particular, members of the *ATL* (*Arabidopsis tóxicos en levadura*) [18], [19] gene family have been shown to be activated by elicitors and to play important roles in defense pathways. The Arabidopsis ATL gene family contains 80 members and is a conserved group of RING zinc-finger proteins that encode putative E3 ubiquitin ligases [20]. *ATL2* and *ATL6* in Arabidopsis and *EL5* in rice, all encoding RING-finger type E3 ligases, have been shown to be rapidly induced in response to the elicitor chitin [18], [21], [22], [23]. Recent work by Hondo et al. [24] demonstrated that the tomato ortholog of Arabidopsis *ATL2*, *LeATL6*, responded to cell wall protein fraction elicitor from the biocontrol agent *Pythium oligandrum* and appeared to regulate the jasmonic acid-dependent defense gene expression. In a screen for chitin-responsive genes in Arabidopsis, we identified an *ATL* family member, *ATL9* (At2g35000; ATL2G), that responded strongly to chitin treatment [6]. Loss-of-function mutations in this gene resulted in increased susceptibility to the powdery mildew pathogen, *Golovinomyces cichoracearum* ( = *Erysiphe cichoracearum*) [6]. Our results here confirm that ATL9 is an E3 ubiquitin ligase and show that it is localized to the endoplasmic reticulum. *ATL9* expression is induced by infection with *G. cichoracearum* and ATL9 function is required for basal defense against this biotrophic pathogen. Interestingly, *ATL9* expression appears to be dependent on NADPH oxidases and mutations in *ATL9* lead to an impairment in the ability of plants to produce reactive oxygen species (ROS) after infection. Expression profiling of *atl9* revealed a complex interplay between chitin-mediated signaling and other defense pathways.

## Results

### *ATL9* (Arabidopsis tóxicos en levadura 9) encodes an E3 ubiquitin ligase with homology to a family of genes induced by wounding and abiotic stress

**Figure 1:**
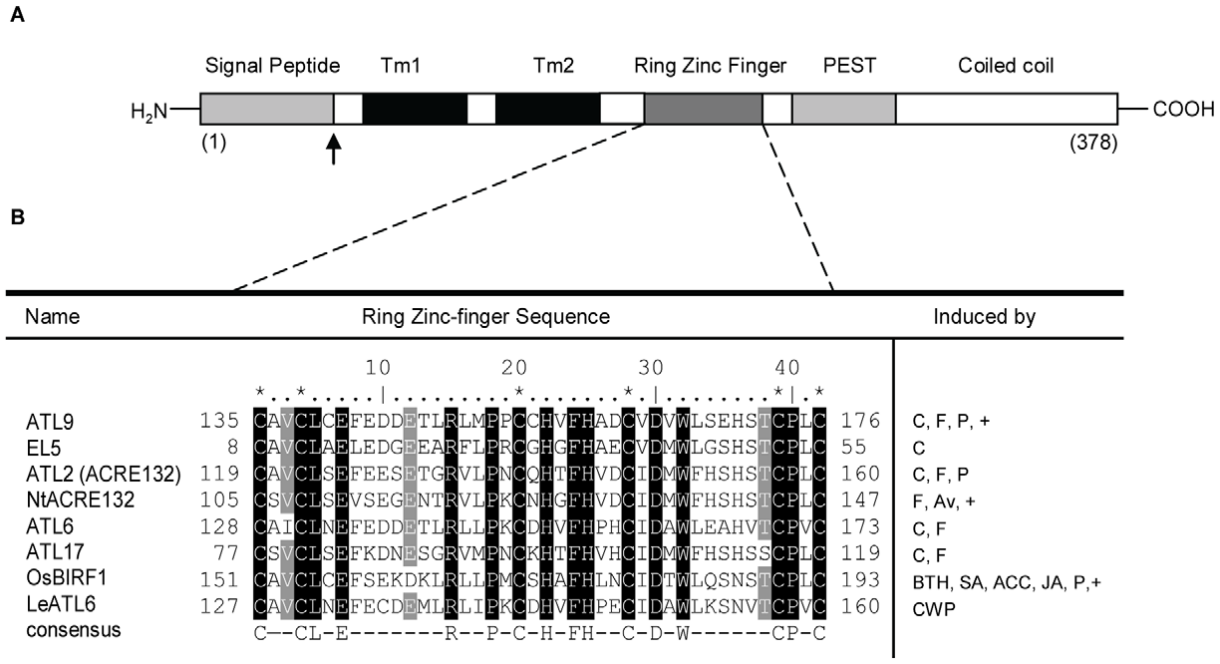
ATL9 structure and sequence alignment between ATL family members. **A**) Schematic diagram of ATL9 protein structure. The protein has an N terminal region (cytosolic), containing a signal peptide (gray); two transmembrane domains (Tm1 and Tm2, in black); and a C3HC3 RING Zinc-finger domain (dark gray), a PEST sequence and a coiled coil domain that extends into the ER lumen (white). **B**) Alignment of ATL9 RING Zinc-finger domain amino acid sequence with the other ATL family members implicated in defense responses in plants. Asterisks indicate conserved cysteines; conserved amino acids are indicated in black, residues conserved in more than an 87.5% are highlighted in gray. The consensus sequence for this group of RING zinc-fingers is: C-X2-CL-X-E-X7-R-X2-P-X-C-X-H-X-FH-X2-C-X-D-X-W-X6-CP-X-C, where X is any amino acid. Induction of the corresponding genes is indicated as: C (chitin), F (flagellin), P (pathogens), Av (Avr9), BTH (benzothaidiazole), ACC (1-aminocyclopropane-1-carboxylic acid), CWP (cell wall protein fraction elicitor), (+) indicates mutants in these genes have been tested for altered pathogen response.

Previous studies by our group have shown that mutants in the gene At2g35000 were more susceptible to fungal infection than wild-type plants [6]. At2g35000 belongs to the *ATL* family [18], [19] of RING (really interesting new gene) zinc-finger proteins and was designated as *ATL9* in a previous review [12]. The ATL9 protein consists of 378 amino acids and contains an N-terminal signal peptide; two predicted transmembrane domains, a C3HC4 RING zinc-finger domain, a PEST domain and a C-terminal coiled coil region (Figure 1A). Three members of the Arabidopsis *ATL* gene family, *ATL2, ATL6* and *ATL17,* are presumed to play a role in defense although their precise functions are unknown at present [21], [25]. Using database searches we identified a total of eight proteins with a high percentage of homology to ATL9, including several ATLs in other plant species such as *Oryza sativa* (*EL5*) [26], *Nicotiana tabacum* (*Avr9*) [27] or Poplar (*PtaRHE1*) [28]. An alignment of the eight protein sequences along with their putative function in defense is shown in Figure 1B. Of the eight proteins, seven are known to be specifically induced by elicitors and four of the proteins (*ATL9, ATL2/ACRE132, ATL6 and ATL17*) are induced specifically by flagellin or chitin (Figure 1B). All eight proteins contain a RING zinc-finger motif with six highly conserved cysteine residues that match the consensus sequence for the C3HC4 type (RING zinc-finger) domain group (http://www.sanger.ac.uk/Software/Pfam/). Of the genes found only three, *ATL9* in Arabidopsis, *NtACRE132* in tobacco and *OsBIRF1* in rice have been tested for their putative role in response to pathogens (Figure 1B).

### The induction of *ATL9* is independent of the classical defense pathways

**Figure 2:**
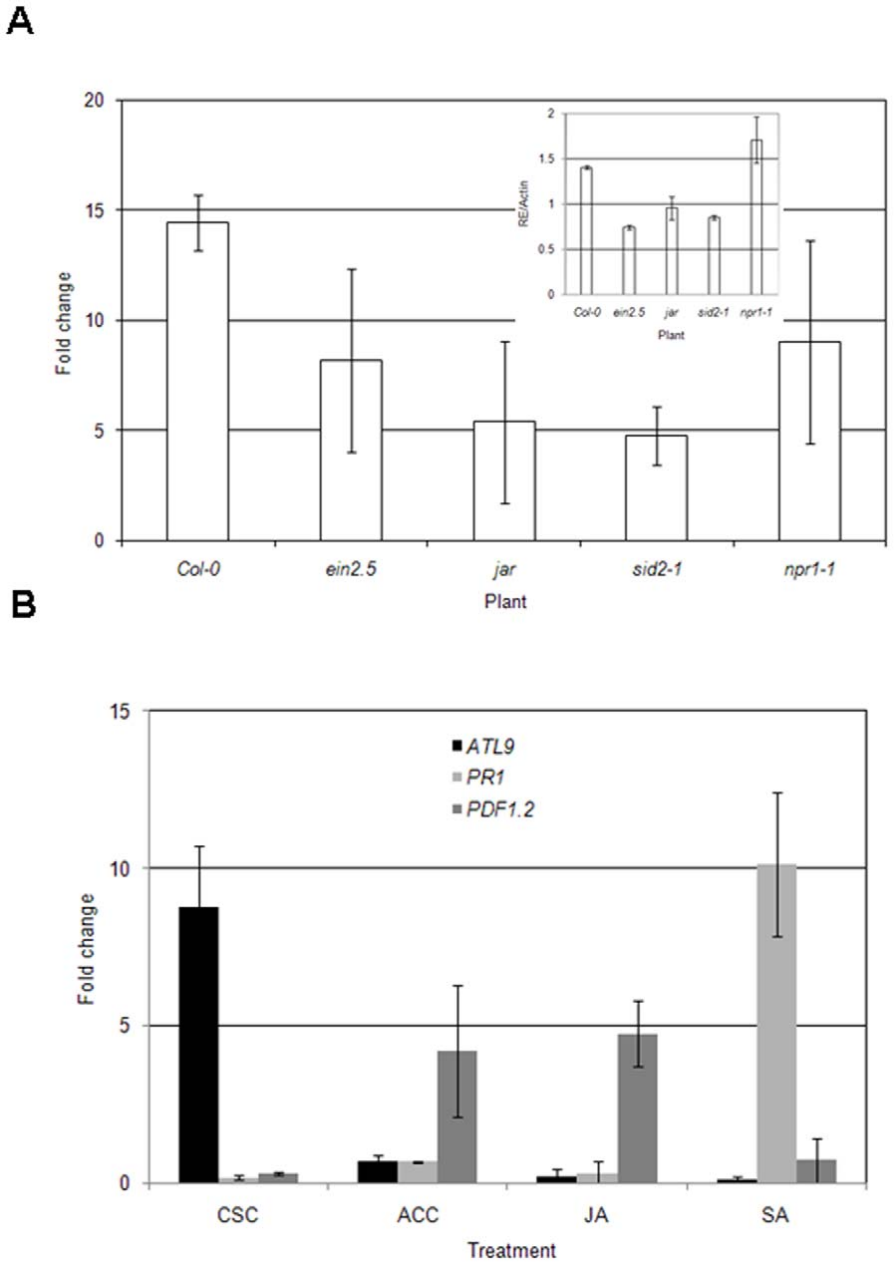
Influence of SA-, JA- and ET-mediated pathways on *ATL9* expression measured by qRT-PCR. **A**) Induction of *ATL9* 30 minutes after chitin treatment in Col-0 wild-type plants and in the *ein2-5*, *jar1 and sid2-1* mutants. **B**) Induction of *ATL9* (black), *PR1* (light gray) and *PDF1.2* (dark gray) in Col-0 plants after treatment with chitin (CSC), 50 µM ACC, 5 µM JA or 0.5 mM SA. Data represent the ratio between mock and treated plants of three independent biological samples. The inset shows relative expression of *ATL9* transcript compared to the actin control (RE/Actin).

Studies have shown that chitin-induced defense responses act through an independent signaling pathway and are not dependent on SA-, JA- or ET-mediated responses [29]. In some cases, however, expression levels of chitin-induced genes were found to be slightly compromised in mutants defective in the SA- and JA-dependent signaling pathways suggesting some level of cross-talk [29]. In order to determine whether the induction of *ATL9* was mediated solely by chitin or might also be regulated by the SA-, JA- or ET-dependent signaling pathways, Col-0 plants and mutants impaired in each signaling pathway (SA: *sid2-1*, *npr1-1*; JA: *jar1*; ET: *ein2-5*) were treated with chitin for 30 minutes and *ATL9* expression was monitored. All plants tested showed induction of *ATL9* when treated with chitin compared to untreated controls, although in all cases levels of induction in the mutant lines were lower than in wild type. Induction of *ATL9* by chitin treatment was higher in Col-0 plants than in any other line tested (Figure 2A). Among the mutants, the highest levels of *ATL9* induction were detected in the *ein2-5* and *npr1-1* mutants, while *ATL9* levels in *sid2-1* and *jar-1* were the lowest (Figure 2A). The basal levels of *ATL9* in each of the mutants are slightly lower or higher than in Col-0 (inset Figure 2A). From these data it appears that the induction of *ATL9* expression by chitin is not dependent on the SA and JA signaling pathways.

To resolve the contributions of each signaling pathway to overall levels of *ATL9* expression, we treated wild type plants with chitin, SA, 1-aminocyclopropane-1-carboxylic acid (ACC) and JA [30], [31]. Expression of *ATL9* and marker genes associated with each pathway (SA: *PR1*; JA- and Ethylene: *PDF1.2*) were quantified 30 minutes after treatment. Only treatment with crab-shell chitin (CSC) induced expression of *ATL9* (Figure 2B). Exposure of plants to ACC, JA and SA resulted in no appreciable expression of the gene (Figure 2B) providing evidence that *ATL9* induction is uniquely associated with chitin-mediated defense responses. As expected, marker genes associated with each of the classical defense pathways were induced by their corresponding signaling molecule (Figure 2B). These results suggest that the SA-, ethylene- and JA-mediated defense pathways do not directly regulate *ATL9* expression but might feedback on the chitin-induced expression of *ATL9*.

### *ATL9* is involved in plant defense against *G. cichoracearum*

**Figure 3:**
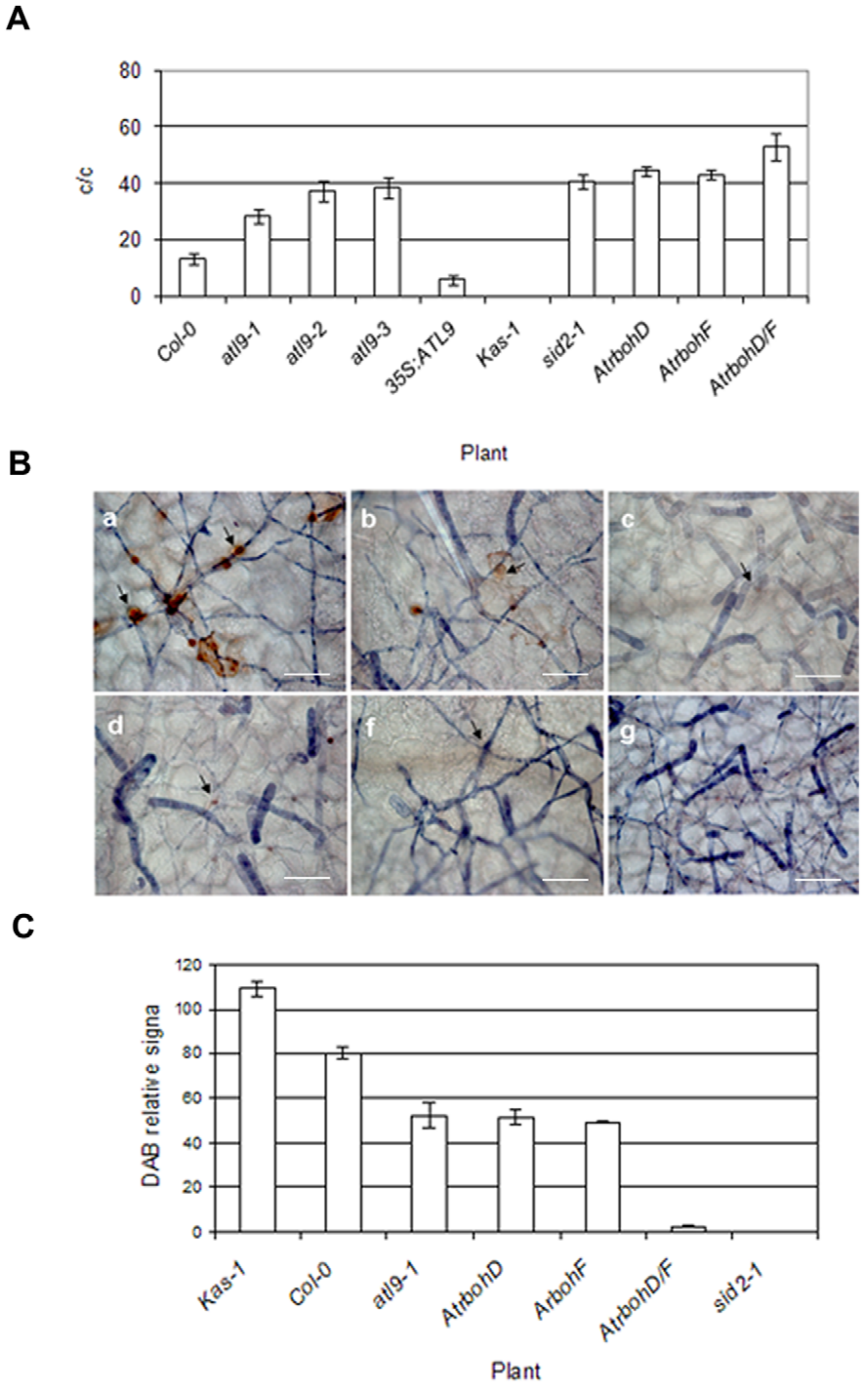
Susceptibility of *atl9* mutants to *Golovinomycetes cichoracearum* and hydrogen peroxide production. **A**) Quantification of *Golovinomyces cichoracearum* growth on Col-0 plants, three different *atl9* T-DNA insertional mutants (*atl9-1-3*), *35S:ATL9*, Kas-1, *AtrbohD*, *AtrbohF*, *AtrbohD/F* and the SA-compromised mutant *sid2-1*. The number of conidiophores per colony (c/c) were counted 6 dpi. Inoculations were performed at a low density (5–10 conidia/mm2; n = 36 plants). Data values represent one of at least three independent experiments with similar results. **B**) Production of hydrogen peroxide on plants infected with *G. cichoracearum*. Cleared leaves of infected plants were stained with trypan blue which stains the fungal hyphae and conidiophores on the leaf surface. Secondary staining with DAB was performed to indicate areas of hydrogen peroxide production (indicated by peroxidase activity). a, Kas-1, b, Col-0, c, *atl9-1, d, AtrbohD, f, AtrbohD/F, g, sid2-1.* Arrows indicate fungal penetration points in epidermal cells with detected hydrogen peroxide production. The images were taken at a final magnification of 40x. Bars.- 5 µm. **C**) Quantification of hydrogen peroxide production in mutants. The intensity of DAB staining was quantified at each inoculated plant and compared to the corresponding uninoculated control to obtain a relative DAB signal. The quantification was performed with the aid of the program Image J.

Mutations in *ATL9* rendered plants more susceptible to infection by the biotrophic fungus *G. cichoracearum* compared to the wild type plants. To more precisely quantify the susceptibility of *atl9* mutants to *G. cichoracearum*, inoculations were performed using three independent *atl9* T-DNA insertional lines, 35S*:ATL9* plants, Col-0, the Kas-1 Arabidopsis accession and *sid2-1* plants. Counts of mature conidiophores 6 dpi showed that all three *atl9* mutants (*atl9-*1, *atl9-2, atl9-3*) supported more than two times the number of mature conidiophores per fungal colony compared to wild type (Figure 3A). The *atl9-1, atl9-2* and *atl9-3* mutations all resulted in a more pronounced susceptibility phenotype than observed in wild type plants. All plants tested were more susceptible than the resistant Kas-1 ecotype and complementation of the *atl9* mutation restored the wild-type defense phenotype (data not shown). Transformed plants containing an over-expression construct of *ATL9* were significantly more resistant to *G. cichoracearum* and showed a 2-fold decrease in conidiophores per colony compared to wild-type plants (Figure 3A).

Since previously published microarray data (NASC array) indicated that expression of *ATL9* in roots was compromised in the NADPH oxidase *AtrbohC* mutant, we tested the defense phenotype of mutants in the NADPH oxidases *AtrbohD* and *AtrbohF* which are expressed in leaves and in the double mutant AtrbohD/F against *G. cichoracearum* (Figure 3A). *AtrbohD*, *AtrbohF* and the SA-compromised *sid2-1* mutant showed comparable numbers of conidiophores per colony and were significantly more susceptible than *atl9*, the *35S:ATL9* line, Col-0 or the resistant Kas-1 accession. The *AtrbohD/F* double mutant was the most susceptible to powdery mildew even surpassing that of *sid2-1* suggesting an additive effect of the individual mutations (Figure 3A).

Because the ROS response is essential for disease resistance we decided to determine if ROS production was compromised in *atl9* mutant and in the *Atrboh* mutants (Arabidopsis thaliana respiratory burst oxidase homolog). Infected leaves of each mutant were double stained with diaminobenzidine (DAB) to visualize H_2_O_2_ and with trypan blue to observe the spread of the fungus 7 dpi with powdery mildew (Figure 3B). Consistent with our hypothesis, H_2_O_2_ production was much lower in *atl9,* compared to that of Col-0 or Kas-1 plants (Figure 3B). H_2_O_2_ localization was focused at points where the fungus was attempting to penetrate the epidermal cells (Arrows, Figure 3B). DAB staining of H_2_O_2_ was not visible in *AtrbohD/F* or the *sid2-1* mutant (Figure 3B). Hydrogen peroxide was detected both at the point of fungal penetration as well as inside some epidermal cells in Col-0 plants (Figure 3B). The highest levels of hydrogen peroxide were detected in the resistant Kas-1 ecotype (Figure 3B) with the highest levels of H_2_O_2_ localizing to points of fungal penetration and in the epidermal cells directly adjacent to penetrated cells. High H_2_O_2_ levels in Kas-1 correlated with a strong inhibition of fungal growth and colony development (Figure 3B). In all cases there was good inverse correlation between the conidiophores per colony (Figure 3A) and the levels of H_2_O_2_ detected in leaves via DAB staining (Figure 3C). Taken together these data indicate that ROS production in epidermal cells is necessary for effective defense against *G. cichoracearum* and this ROS production is impaired in the atl9 mutant, *AtrbohD* and *AtrbohF*. This provides further evidence that functional *ATL9*, *AtrbohD and AtrbohF* expression are needed for effective defenses against powdery mildew.

### Basal expression of *ATL9* and induction by chitin depends on NADPH oxidase activity

**Figure 4:**
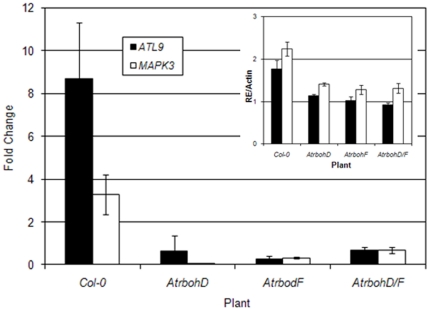
The basal expression of *ATL9* and its induction by chitin depends on NADPH oxidases. Plants were treated with chitin for 30 minutes and harvested for analysis. Expression levels of *ATL9* were measured by qRT-PCR. The inset shows the relative expression of *ATL9* transcript compared to actin (RE/Actin) in control plants. Data represent the ratio of expression between mock and treated plants of three independent biological samples.

The production of reactive oxygen species (ROS) is a key characteristic of the initial defense response of plants to pathogen attack and chitin treatment is known to elicit ROS production in roots although not in leaves [32]. Several NADPH oxidases in Arabidopsis have been identified and their roles in ROS generation and plant defense characterized [33], [34], [35]. A search of publically available microarray data (http://affymetrix.arabidopsis.info/narrays/geneswinger.pl) revealed that expression of *ATL9* in roots was repressed by 50% in the *AtrbohC* mutant [36] compared to wild-type plants. *AtrbohC* plants are also more susceptible to the powdery mildew pathogen *G. cichoracearum* (data not shown). These data raised the possibility that the induction of *ATL9* by chitin might be dependent on NADPH oxidase activity. To test this hypothesis we treated Col-0 plants and three mutants impaired in NADPH activity (*AtrbohD*, *AtrbohF*, *AtrbohD/F*) with chitin and analyzed the transcript levels of *ATL9* by qRT-PCR (Figure 4). The *MAPK3* gene was used as a control since it is known to be induced by chitin [29]. Though *ATL9* was induced by chitin in Col-0 plants, its induction was significantly reduced in the three *Atrboh* mutants (Figure 4). *ATL9* was still expressed in untreated *Atrboh* mutants but at half of that observed in untreated wild-type plants (inset Figure 4). These data indicate that basal expression levels of ATL9 are dependent on *AtrbohD and F*.. A similar pattern of reduced induction and reduced basal expression was observed for the *MAPK3* gene in all the lines tested (Figure 4 and inset). These results indicate that both basal transcription levels of *ATL9* as well as the induction of *ATL9* by chitin depend on NADPH oxidase activity. These results are in line with previous NASC array data showing that *ATL9* transcription levels are impaired in the *AtrbohC* mutant.

### *ATL9* is induced by *G. cichoracearum* and has a complex pattern of expression post-inoculation

**Figure 5:**
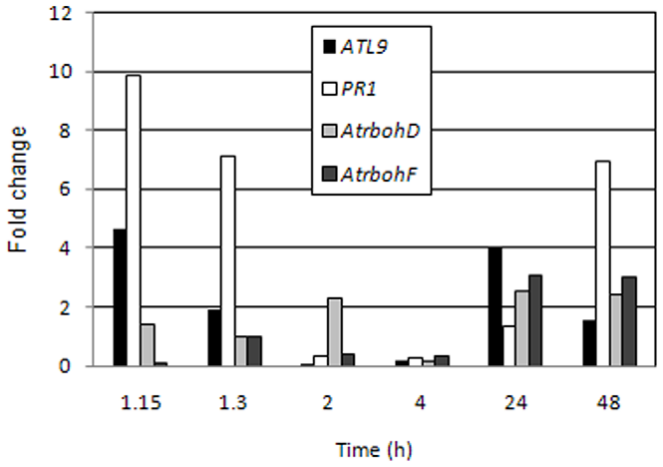
Relative expression levels of selected genes at various time points after inoculation with *Golovinomyces cichoracearum*. Col-0 plants were inoculated with *G. cichoracearum* at high concentration. qRT-PCR data represent the ratio between mock and treated plants of three independent biological samples.

Since *atl9* mutants are more susceptible to *G. cichoracearum* (Figure 3A) than wild-type plants and *ATL9* basal expression and induction by chitin is dependent on NADPH oxidases (Figure 4 and inset), we were interested in determining the timing of *ATL9* expression after fungal infection. Expression levels of *ATL9*, *AtrbohD*, *AtrbohF*, and the defense marker genes *PR1* and *PDF1.2* were monitored using qRT-PCR at several time points after infection with powdery mildew [37], [38]. Wild-type plants were heavily inoculated with *G. cichoracearum* and tissue samples were taken at four early time points: 1 hr., 1.5 hr., 2 hr. and 4 hr. post-infection and at two late infection time points; 24 hr. and 48 hours post-infection (hpi). Quantitative analysis of the gene expression revealed that *ATL9* is induced very early in the infection process (1 hr.; Figure 5) and then expression quickly drops off to negligible levels at 4 hpi in a pattern similar to that of *PR1* expression (Figure 5). *ATL9* expression again rose in the later time points (24 and 48 hpi). By 24 hpi, ATL9 expression was again induced to levels similar to those observed in the 1 hour time point (Figure 5). *AtrbohD*, *AtrbohF*, and *PDF1.2* were not significantly induced early in the infection process but were present at higher levels in the later stages of the infection beginning at 24 hpi (Figure 5). These data may indicate that an early step in pathogen recognition or the infection process is critical for activation of *AtrbohD*, *AtrbohF*, and *PDF1.2*.

### ATL9 has ubiquitin ligase activity

**Figure 6:**
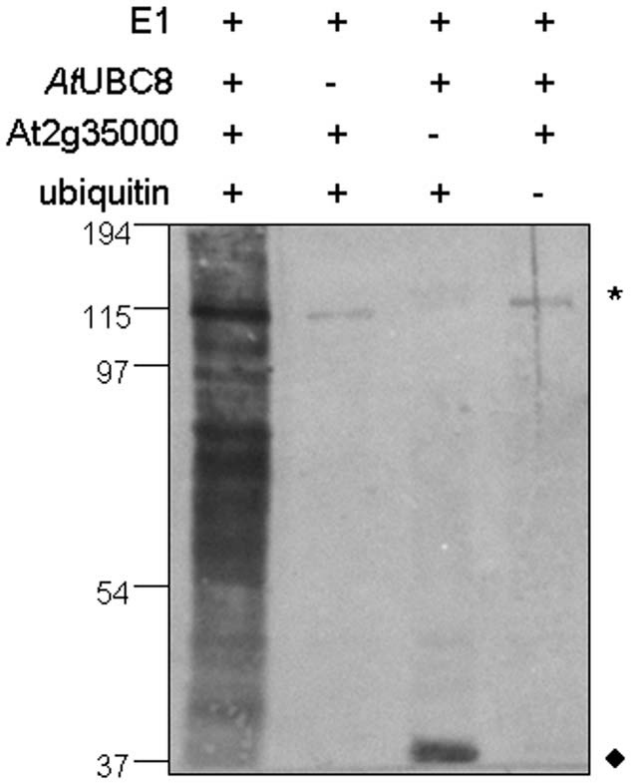
ATL9 is capable of mediating protein ubiquitination in a E2-dependent manner. The complete *in vitro* ubiquitination assay (lane 1) contained recombinant yeast E1 enzyme, recombinant 6x histidine-tagged Arabidopsis E2 enzyme UBC8, GST-tagged ATL9 and ubiquitin. Omission of *At*UBC8 (lane 2), GST-ATL9 (lane 3) or ubiquitin (lane 4) from the assay resulted in a loss of protein polyubiquitination as indicated by a lack of the ubiquitinated proteins compared to the complete assay (lane 1). Ubiquitinated proteins were visualized by western blot analysis using anti-ubiquitin antibodies. * Indicate non-specific cross-reactive proteins. ♦ Represent *At*UBC8-Ub(n). Molecular weight markers (kDa) are shown to the left of the blot.

The RING zinc-finger domain is known to be essential for the function of ubiquitin E3 ligases [39] and two other members of the ATL family, ATL2 and ATL6, have been identified as E3 ligases in Arabidopsis [18], [21]. In order to determine whether ATL9 might function as an E3 ligase, we analyzed ATL9 activity using an *in vitro* ubiquitination assay (Figure 6). A GST:ATL9 fusion protein was affinity purified and its E3 ligase activity was assayed *in vitro*. Multiple forms of ubiquitinated proteins were detected in the complete assay by the anti-ubiquitin antibodies (Figure 6, lane 1). The omission of AtUBC8 (Figure 6, lane 2), GST-ATL9 (Figure 6, lane 3) or ubiquitin (Figure 6, lane 4) from the assay resulted in a loss of protein polyubiquitination (Figure 6, lane 1) confirming that ATL9 has ubiquitin ligase activity.

### The ATL9 protein is localized to the Endoplasmic Reticulum (ER)

**Figure 7:**
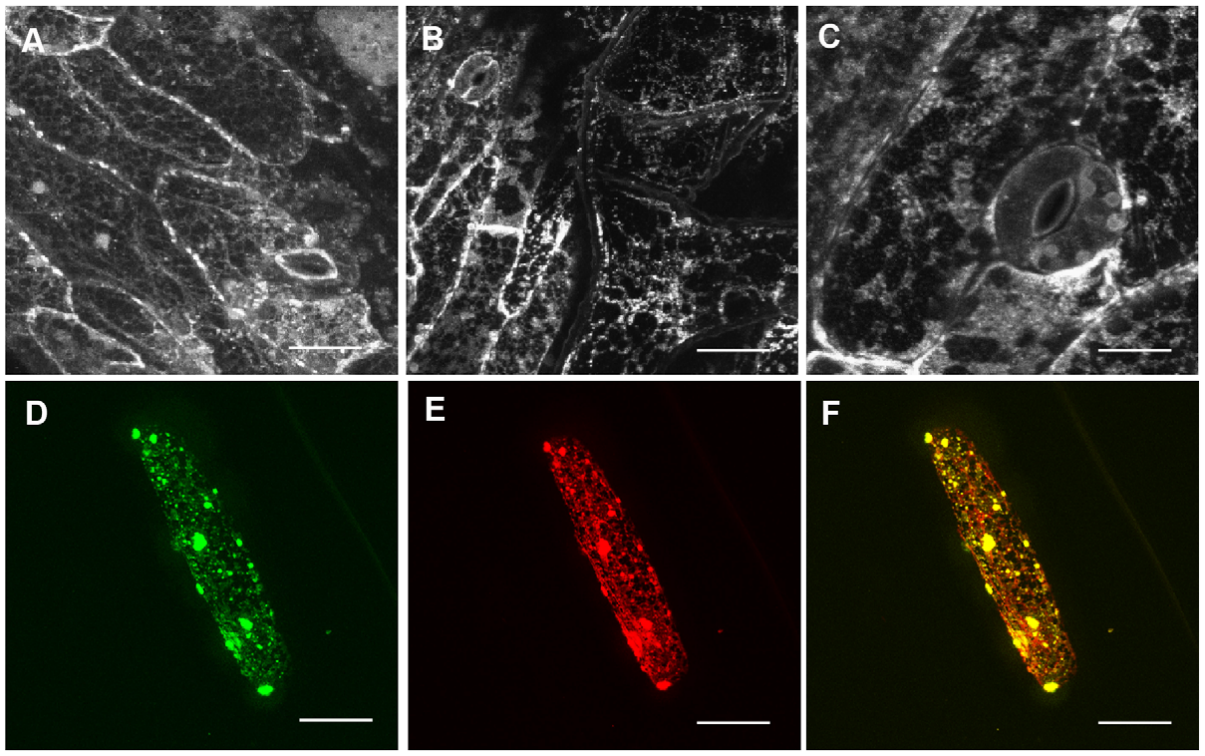
The ATL9 protein localizes to the ER. **A–C**) Confocal images of leaf epidermal cells in transgenic Arabidopsis plants expressing *ATL9p*:*ATL9*:GFP showing protein localization to the endoplasmic reticulum. **B**) Confocal image of a leaf trichome showing *ATL9p*:*ATL9*:GFP localizing to the ER. **D–F**) Co-localization of the 35S:*ATL9*:GFP fusion and ER-rk marker. Onion epidermal cells were co-bombarded with 35S:*ATL9*:GFP and ER-rk and visualized using fluorescence microscopy. D) 35S:ATL9:GFP. E) ER-rk (mCherry). F) GFP/mCherry overlay. Bars: 3 µm (A,B), 1 µm (C), 10 µm (D-F).

Defense-related proteins can be found in a diverse array of cellular compartments. To determine the localization of ATL9, a construct was made containing a C-terminal fusion of the green fluorescent protein (GFP) to the *ATL9* gene under the control of its native promoter (*ATL9p*:*ATL9*:GFP). Stable transgenic lines were created by transforming Col-0 plants with *Agrobacterium tumefaciens* containing the *ATL9p*:*ATL9*:GFP construct. Upon visualization, fluorescent signal was detected in the endoplasmic reticulum (ER) of leaf epidermal cells (Figure 7A–C). Localization of ATL9 was observed in the ER of all tissues tested and was particularly high in roots (Figure 7A–C; data not shown). For more precise localization a second construct containing a fusion of GFP to the C-terminus of *ATL9* driven by the CaMV 35S promoter (35S:*ATL9*:GFP) was co-bombarded into onion epidermal cells with a known ER luminal marker ER-rk (signal peptide of *AtWAK2*:mCherry:HDEL) [40]. When visualized with fluorescent microscopy, 35S:*ATL9*:GFP co-localized with the ER-rk marker in the onion epidermal cells confirming its localization to the ER (Figure 7D–F). In parallel we also transiently expressed an N-terminal construct of ATL9, 35S:GFP:*ATL9,* in tobacco with a 35S:GFP construct as an internal negative control for GFP localization to the nucleus. Again, *ATL9* was expressed transiently in the ER of tobacco epidermal cells confirming our results in Arabidopsis and onion (Figure S1A). Fluorescence was detectable within the nucleus in the tobacco cells expressing the 35S:GFP construct and the GFP signal could be seen in the ER surrounding the nuclear membrane (Figure S1B). A western blot with the protein extract from the transiently transformed tobacco tissue was performed and hybridized with anti-GFP antibody to confirm the size of the ATL9 and GFP fusion proteins (Figure S1C).

Since some PAMP response related proteins are transported to the vesicle after stress responses [41], we wanted to determine if ATL9 responded in a similar manner. To determine if ATL9 localization changed after elicitor treatment, transgenic plants expressing *ATL9p*:*ATL9*:GFP were treated with chitin (100 mg/L) by direct infiltration of the leaves and observed at five minute intervals for one hour. No change in ATL9 localization was detected regardless of the time after treatment with chitin (data not shown).

### Microarray expression analysis of *atl9*

**Figure 8:**
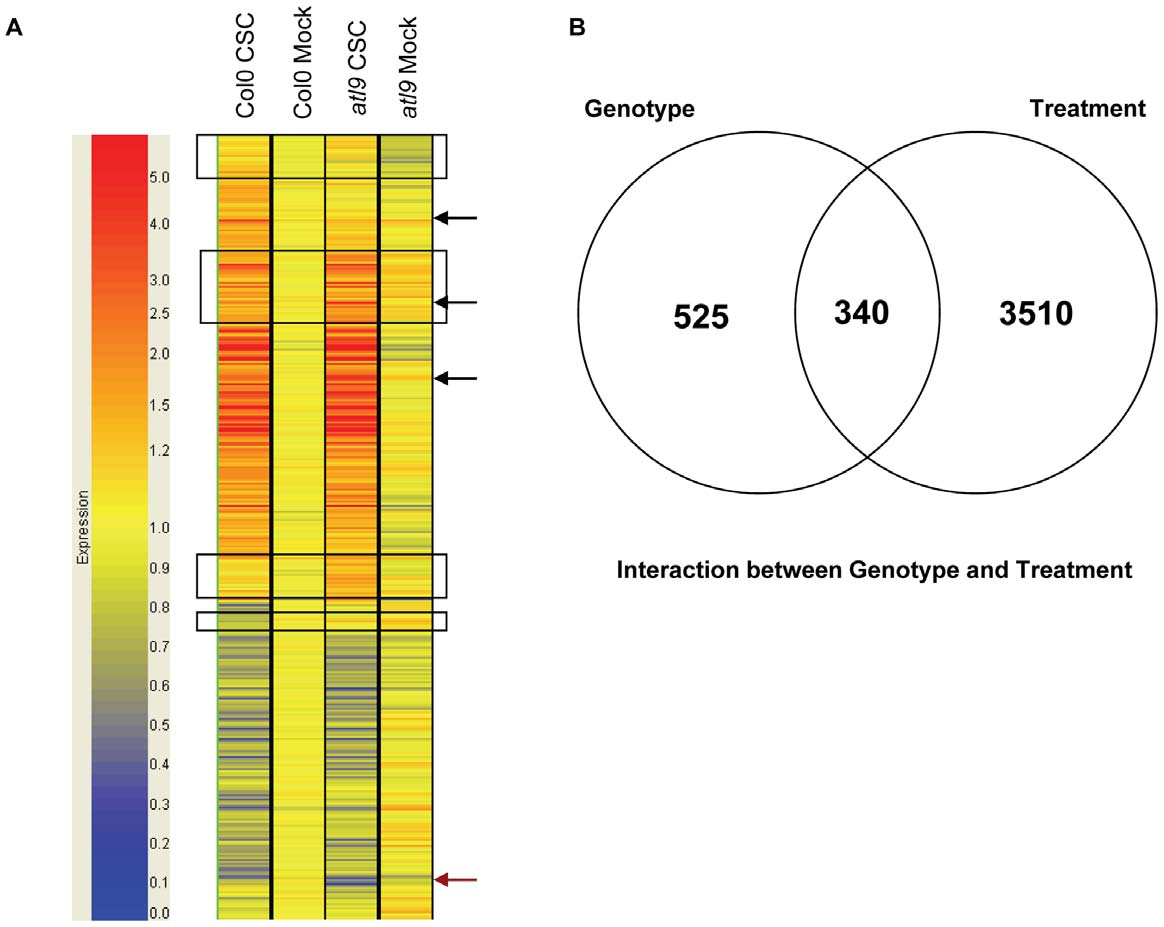
Microarray analysis of *atl9* mutant after chitin treatment. **A**) Hierarchical cluster of ratio values (relative to the water control treatment) of 16,530 genes analyzed in Col-0 wild-type and *atl9* plants treated with crab shell chitin (CSC) for 30 minutes. Each gene is represented by a single row and each column represents an individual treatment. Red represents up-regulated genes; blue, down-regulated genes; and yellow, genes with no change in expression. Groups of genes expressed differentially are delineated with rectangles. Three genes (black arrows) were changing due to interactions between treatment and genotype and one gene (red arrow) was genotype interaction specific. **B**) Venn diagram representation of results from hierarchical clustering. A total of 4,375 genes were differentially expressed between genotypes and treatments. The statistic used for clustering was two-way ANOVA. Genotype and treatment groups were analyzed using a p-value of 0.5 with p-value >0.5 =  not significant; p-value <0.5 =  significant.

To identify genes whose expression is dependent on *ATL9* function, Col-0 plants and *atl9* mutants were treated with chitin for 30 minutes and their expression profiles were examined using Affymetrix ATH1 arrays. Of the 22,677 genes represented, 16,530 were defined as present and of these 4,375 genes showed altered expression in the *atl9* mutant compared to wild-type plants (Figure 8A and 8B). Genes were divided into (1) those that exhibited genotype-specific differences in expression between wild type and *atl9* and (2) those that responded to treatment with chitin. Figure 8B shows that the majority of genes that exhibited altered expression responded to chitin treatment regardless of plant genotype (3510), while a smaller number (525) fluctuated between wild-type plants and the *atl9* mutant irrespective of treatment. An intermediate number of genes (340) were changed both by the loss of *ATL9* expression and by chitin treatment (Figure 8B). Data on selected genes within each category are given in Table S1.

Several genes with known roles in plant defense were identified that were specifically altered between wild-type plants and *atl9* mutants (Table S1). Two genes associated with SA-mediated defense responses, *PR-1* and *SID2/ICS1* were repressed in plants lacking *ATL9* (Table S1). *PR-1* levels (At2g14580) were decreased 3-fold in the *atl9* mutant compared with Col-0 plants. This finding is surprising since we have shown that *PR-1* gene expression is induced by infection with *G. cichoracearum* but not by chitin-treatment alone (Figure 5, Table S1). Expression of *SID2/ICS1* (isochorismate synthase; At1g74710) was slightly down-regulated in the *atl9* mutant (0.77-fold) compared to Col-0 though in both cases the gene was induced by chitin. *PEN3* expression was reduced by 50% in the *atl9* mutants in contrast to *PEN1* and *PEN2* whose expression was unchanged (Table S1). Expression of *PDF2.5*, an antimicrobial peptide, was slightly lower in *atl9* plants compared to Col-0. However, when treated with chitin *PDF2.5* was strongly induced in wild-type plants but its expression remained unchanged in the *atl9* mutant (Table S1). Similarly the *PCC1* gene (pathogen and circadian controlled 1) was down-regulated in response to the loss of *ATL9*. As in the case of *PDF2.5*, *PCC1* was strongly up-regulated in Col-0 plants upon chitin treatment and was down-regulated in the *atl9* mutant (Table S1).

Most of the defense genes surveyed were not significantly affected by the loss of *atl9* expression and exhibited similar responses to chitin-treated control plants (Table S1). However, several defense genes had enhanced expression levels after chitin treatment in the *atl9* mutant including *PEN1*, *PAD4*, two disease resistance-like proteins, *ACRE1b*, *ATL17* and protein phosphatase 2C (Table S1). Interestingly, *AtrbohD* expression was induced to higher levels in chitin-treated *atl9* compared with wild-type controls. Similar increases were not observed in the expression of *AtrbohC* or *AtrbohF*.

### Defense genes associated with elicitor-mediated responses

To identify defense-related genes that were specifically linked to the chitin signaling pathway, we compared the expression of genes known to be induced by both abiotic and biotic stresses in chitin-treated Col-0 wild type and *atl9* plants (Table S1). We first analyzed the expression levels of the *ATL9* family members *ATL2-ACRE132*, *ATL6* and *ATL17* (Table S1). In all cases, the *ATL* family members were induced at significantly higher levels in wild-type plants, compared to *atl9* mutants. This strongly suggests that ATL9 is important in initiating the expression of genes involved in chitin-mediated responses. We were also interested in determining the responses of the known elicitor receptors, CERK1 (LysM RLK1), FLS2, BAK1 and EFR in the *atl9* mutant. The induction of the gene encoding the chitin receptor LysM RLK1 was essentially unchanged in the *atl9* mutant and wild-type plants. Surprisingly, expression levels of the flagellin receptor gene, *FLS2* were strongly induced by chitin in Col-0 plants but not in the mutant (Table S1), suggesting that induction of *FLS2* may be dependent on *ATL9* expression. Expression of *BAK1* which acts as a co-receptor of flagellin with FLS2 [9], [42] and the bacterial EF-Tu receptor EFR [7] were both induced by chitin treatment but at similar levels in both the *atl9* mutant and in wild-type plants. In addition, *ACRE1b*, a gene highly induced by treatment with *flg22* [5] was also strongly induced by chitin treatment in wild-type plants. Taken together these results indicate that activation of elicitor-mediated signaling by chitin and flagellin are closely intertwined in plants.

To better understand the interactions between chitin-mediated defense signaling and signaling mediated through SA, JA and ET the expression levels of key genes in each of the signal transduction pathways were monitored (Table S1). The induction of *MAPK3* and *MAPK5*, both known to be important in signaling pathways initiated by flagellin [43] were very similar in *atl9* and Col-0 plants indicating that these MAP kinases are upstream of ATL9 activity or are working in an independent signal transduction network. A similar induction by chitin in both the *atl9* mutant and wild-type was also observed for *MAPKK4*. Other genes involved in SA-, JA- and ET-mediated signaling (*RAR1, SGT1, HSP90, COI1, ETR1, EIN3, PAD2, JAR1 or SAG101*) showed no differences in expression levels in either chitin-treated control or *atl9* plants or in mock-treated plants pointing to the fact that these genes are not influenced by *ATL9* expression. However, the expression of the lipoxygenase-3 (*LOX3*) gene, which is involved in octadecanoid biosynthesis leading to the production of JA in plants, was highly induced in both *atl9* and Col-0 plants by chitin.

## Discussion

### ATL9 is an E3 ubiquitin ligase that is integral to defense against fungal pathogens

Recent work has highlighted the ubiquitin-proteasome system (UPS) and its associated E3 ubiquitin ligases as regulators of the plant defense response and it is clear that these proteins play an important part in disease resistance [12], [44]. In the current study we have shown that ATL9 is a RING-type E3 ubiquitin ligase strongly induced by chitin. The *ATL* gene family encodes a group of proteins that share three specific characteristics: 1) rapid induction (<1 hour) after elicitor treatment, 2) a highly conserved RING-H2 zinc-finger domain with at least six cysteines and three histidines conserved and 3) at least one amino-terminal transmembrane domain. Most members of the *ATL* gene family are predicted to function as single subunit E3 ubiquitin ligases with seventeen members of the ATL family known to be expressed in Arabidopsis [25]. Although determination of a common function for them is still in progress, mutations in members of the ATL family have been shown to have an altered defense response to pathogens [25]. The *ATL2* gene was shown to be specifically induced by chitin but not by other elicitors of classic defense pathways [18]. Constitutive over-expression mutants of *ATL2* induced high levels of pathogen-related genes such as *NPR1-1* and the phenylpropanoid biosynthetic enzymes phenylalanine ammonia lyase and chalcone synthase [21], [45]. The *EL5* gene in rice is also a member of the ATL family and is rapidly and transiently induced by chitin [23], [26]. EL5, like ATL9, is an E3 ubiquitin ligase and is hypothesized to play a role in defense responses through protein turnover via the UPS [22]. Additionally, the *ACRE-132* gene, an *ATL* protein from tobacco has been shown to be induced during the defense response mediated by the interaction between Avr9 and Cf9 in the response to the fungal pathogen *Cladosporium fulvum* [46].

ATL9 is unique among ATL family members that have been characterized since it contains a PEST (Pro-Glu-Ser-Thr) domain. PEST domains are common in proteins that are rapidly degraded in the cell [47] and are suggested to serve as proteolytic signals [48] involved in ubiquitination and internalization of proteins. While few studies in plants have examined the role of PEST domains and the UPS in defense, there are numerous studies in mammals describing PEST domains and their role in defense against cellular pathogens [49]. The presence of a PEST domain in the ATL9 protein may imply that there are multiple ways to control its expression and that ATL9 may play a critical role in plant defense similar to that of the Mcl family of genes in humans which are necessary for cell survival in the immune system [50]. In plants, the tomato *Ve* gene contains a PEST domain that is necessary for defense against *Verticillim* species [51].

Our data show that ATL9 is necessary for resistance against *G. cichoracearum* (Figure 3A). *ATL9* transcription levels appear to be tightly regulated; after chitin treatment the gene is induced within 30 minutes [6] and in plants infected with *G. cichoracearum ATL9* expression can be detected in less than one hour by qRT-PCR (Figure 5). We hypothesize that *ATL9* expression is also down-regulated quickly (<1 hour) with ATL9 binding its target for elimination via the UPS followed by rapid ATL9 degradation via its PEST domain. Over-expression of *ATL9* produced plants that were more resistant than wild-type (Figure 3A) suggesting that the gene is degraded rapidly under normal conditions in wild type plants post-infection. Further experiments with deletion constructs in the PEST domain of *ATL9* will be useful in characterizing *ATL9′s* regulation and in confirming our hypothesis regarding its degradation.

### ATL9 appears to be involved in ER stress responses and ERAD during innate immunity

In 2003, Takemoto et al. showed cytoplasmic aggregation and accumulation of ER and Golgi bodies around the sites of fungal penetration [52]. These cellular rearrangements suggest that the production and secretion of plant materials are activated around sites of infection or penetration. Transgenic plants expressing an *ATL9p:ATL9:*GFP fusion construct showed that ATL9 was localized to the ER membrane (Figure 7A–C). Localization of the ATL9 protein at the ER membrane not only allows the protein to remain close to the site of infection/penetration but also places the protein where it can be quickly degraded after carrying out its E3 activity. We hypothesize that ATL9 may attach ubiquitin to either plant- or fungal-derived proteins that are active near sites of infection. Several important genes in plant defense have been shown to localize to the ER. The Cf-9 resistance gene, which confers resistance to *Cladosporium fulvum* races expressing the Avr-9 avirulence gene, contains a C-terminal di-lysine motif (KKXX) targeting Cf-9 to the ER [53]. ACRE-132, also a member of the ATL family, has been shown to be induced during Avr-9/Cf-9 interaction and may be an active player in that response [46]. The barley HSP90 protein is localized to the ER plasma membrane like ATL9 [54] and has been shown to interact with RAR1 and SGT1, two genes involved in R-gene mediated resistance to fungi, in yeast two-hybrid experiments. HSP90, RAR1 and SGT1 are hypothesized to form a chaperone complex that mediates the folding of R-proteins and their incorporation into functional complexes. Data supporting the role of an HSP90-RAR1-SGT1 complex as a chaperone active in R-gene mediated defenses have been shown in both barley and Arabidopsis [44], [55], [56]. Though our understanding of the role of ATL family members in defense is incomplete, it is intriguing to speculate that ATL9 or other family members may form complexes similar to HSP90-RAR1-SGT1 that are necessary for defense against *G. cichoracearum*.

E3 ligases in the ER are often associated with endoplasmic reticulum associated degradation (ERAD) a process involved in targeting misfolded proteins for ubiquitination and subsequent degradation by the proteasome [57], [58]. In the ER, the ATL9 protein would have its RING domain and PEST domain exposed to the ER lumen. We hypothesize that ATL9 acts to ubiquitinate a negative regulator of plant defense responses in the ER lumen or some unknown luminal protein that is not properly folded during stress-induced ERAD. The ATL9 protein shares homology with the well-characterized ERAD E3′s Hrd1 and gp78 [59]. Both Hrd1 and gp78 are known to be induced during cellular stress and participate in the unfolded protein response. The yeast Hrd1 protein is at the center of a large protein complex involved in the ubiquitylation of ER luminal and membrane proteins. Since ATL9 is an ER membrane resident protein whose RING domain projects into the ER lumen, it is interesting to speculate that ATL9 may also function in a group of proteins similar to the Hrd1 complex that is involved in ERAD induced by biotic stresses. Further experiments directed towards identifying both ATL9 interacting partners and its' protein target will be helpful in understanding its precise role.

### Expression and chitin *ATL9* gene response depends on NADPH oxidases

The oxidative burst and the production of ROS is an essential component of plant defense. This pathway is mediated by the activation of the NADPH complex which is necessary for the production of H_2_O_2_. Following H_2_O_2_ production, several downstream signaling events are initiated including calcium mobilization to the cytoplasm, protein phosphorylation, MAP kinase activation and defense gene expression [53], [60]. In Arabidopsis, the NADPH family of oxidases consists of ten members (*AtrbohA* –*AtrbohJ*) which are homologs of gp9^phox^, a subunit of the mammalian NADPH oxidase complex [33]. Infection studies of Arabidopsis mutants in *AtrbohD* and *AtrbohF* have shown that *AtrbohD* is responsible for reactive oxygen species produced after infection with avirulent bacteria or oomycete pathogens whereas *AtrbohF* is integral to the regulation of the hypersensitive response [61]. In the current study, we demonstrate that the induction of *ATL9* by chitin is dependent on both *AtrbohD* and *AtrbohF* and that MAPK3 activation is also dependent on *Atrboh* expression. These results suggest that the early steps in chitin recognition are reliant on *Atrboh* functionality (Figure 3). Our data also demonstrate that both *AtrbohD* and *AtrbohF* mutants are more susceptible to *G. cichoracearum* than wild type plants (Figure 3A–B), indicating that *Atrboh* activity is necessary for defense against powdery mildew.

Recent work [62] has demonstrated that *Atrboh* genes can modulate and antagonize SA-dependent cell death signals. ROS produced by *AtrbohD* limited the spread of the cell death induced by the bacterial pathogen *P. syringae DC3000* and the necrotroph *B. cinerea*. Our current study clearly shows that both *ArbohD*, *AtrbohF* mutants were more susceptible than wild type to *G. cichoracearum* and those quantitative results are directly correlated with the low levels of hydrogen peroxide accumulation at the point(s) of penetration detected in these mutants (Figure 3B–C). Hydrogen peroxide production in *AtrbohD, AtrbohF,* and *AtrbohD/F* as well as in the *atl9* mutants was essentially half of that in wild-type plants. Interestingly the Kas-1 accession [63] that is resistant to *G. cichoracearum* had increased levels of peroxide (Figure 3C) at sites of attempted penetration (Figure 3B) although no cell death was observed in these cells and plants were phenotypically resistant. These data imply that careful regulation of hydrogen peroxide production in Kas-1 plants is sufficient to inhibit fungal penetration without cell death [62] suggesting that salicylic acid may activate defense signaling in cells that are spatially removed from infection sites without activating cell death. Torres et al. [62] further hypothesize that *Atrboh*s are necessary for the suppression of unwanted cell death in cells where increased levels of SA are required to activate defense responses. *G. cichoracearum* resistance in Kas-1 plants may be dependent on *Atrboh's* ability to induce higher levels of H_2_O_2_ production at sites where the fungus is attempting to penetrate epidermal cells while simultaneously repressing cell death in those same cells. This hypothesis is supported by the fact that *Atrboh* mutants had virtually no peroxidase activity at penetration sites and were much more susceptible to *G. cichoracearum* (Figure 3A–B). Although a connection between the *Atrboh* genes, chitin induction of *ATL9* and resistance to *G. cichoracearum* remains to be definitively established, our results clearly show that NADPH oxidase activity is required for *ATL9* induction after chitin recognition. This is in line with our results showing that the expression levels of *ATL9* are impaired in both mutants compared with the wild type plants (inset Figure 4B),as in root tissues of *AtrbohC*. The activity of AtrbohD, AtrbohF and ATL9 are needed for effective defense against powdery mildew. This study provides evidence for the first time that AtrbohD and AtrbohF activity, in plant leaves, are required for *G. cichoracearum r*esistance and for some chitin-elicited defense responses.

### Possible mode of action of ATL9 in chitin recognition and chitin-mediated innate immunity

**Figure 9:**
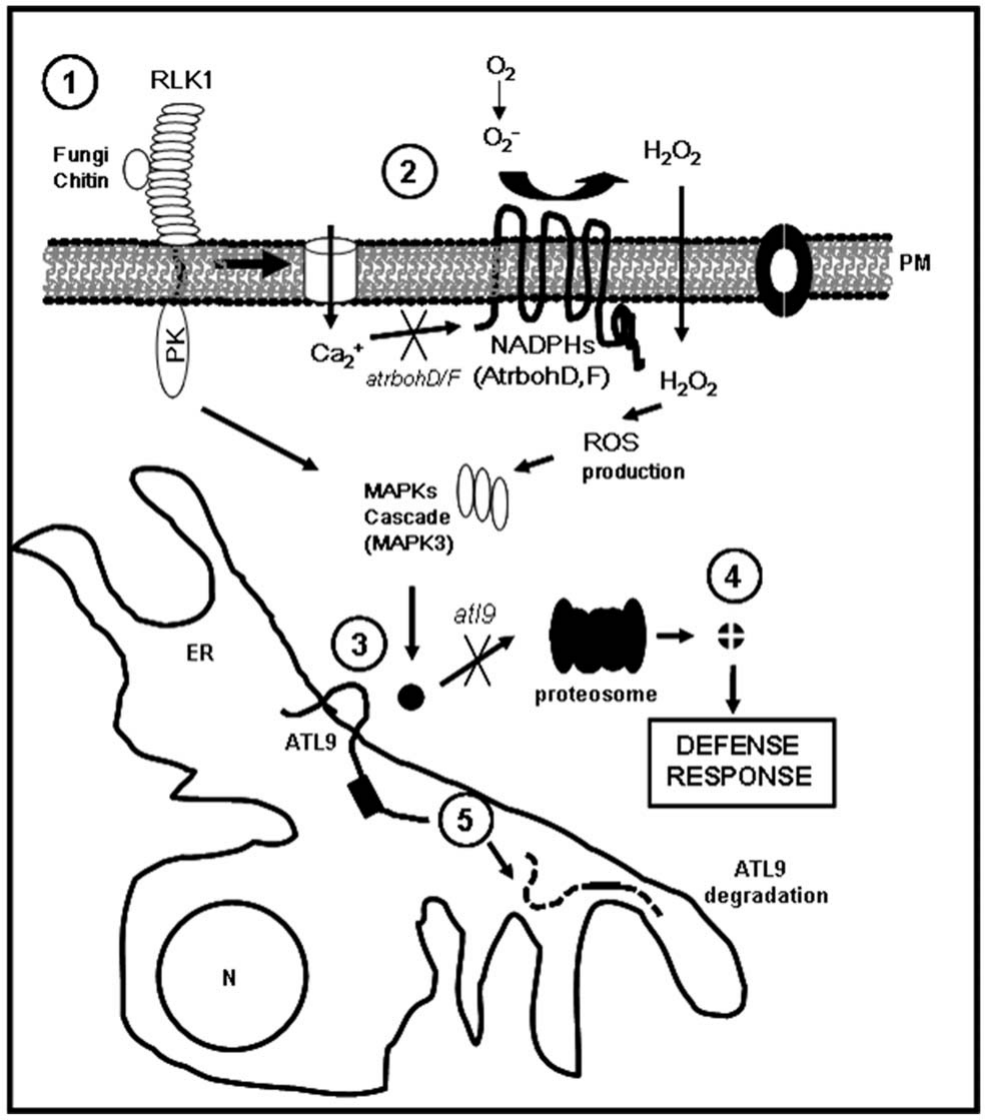
Proposed role of ATL9 in chitin-NADPH mediated innate immunity. 1. Recognition of chitin released from fungal cell wall by LysM-RLK1 receptor. 2. Increase of intra-cellular calcium leading to the activation of the NADPH oxidases AtrbohD and F at the membrane initiating the production of ROS and activation of a MAPK cascade(s). 3. Induction of ATL9 and insertion into the ER membrane. We propose that ATL9 is involved tagging an inhibitor of plant defense for degradation by the proteosome 4. Defense response to fungal pathogen is activated after inhibitor degradation. 5. The ATL9 protein is rapidly degraded in the cell by either the proteosome pathway or a calcium-mediated pathway (similar to the mammalian protein calpain).

We hypothesize that upon chitin recognition/fungal infection intracellular calcium levels increase leading to the activation of Arabidopsis NADPH oxidases (Figure 9). The subsequent production of ROS and activation of MAPK signaling cascades induce *ATL9* expression and its transport to the ER membrane. Here ATL9, either alone or working in a complex would ubiquitinate its target protein(s) for degradation and appropriate defense responses would be activated. After ubiquitylation of its target protein, ATL9 is rapidly degraded via its PEST domain (step 5, Figure 9). A similar mechanism to the one we propose has been described during recognition of human PAMPs [64]. We predict that the target of ATL9 is a negative regulator of defense responses since *atl9* mutants are more susceptible to fungal pathogens. In these mutant plants, we suspect that the negative regulator cannot be ubiquitinated upon receipt of appropriate signals and thus defense responses cannot be activated (Figure 9). Our data clearly indicate that ATL9 is an E3 ligase that is essential for defense against powdery mildew and requires the expression of NADPH oxidases for its activity. This study is the first to directly implicate an ATL family member in chitin responses mediated by NADPH oxidase activity. Future studies identifying ATL9′s targets and its possible interacting partners will be integral in order to better assess its role in innate immunity and defense against fungal pathogens.

## Materials and Methods

### Biological Materials

*Arabidopsis thaliana* ecotype Columbia (Col-0) was used as the control in all experiments. All mutants and transgenic plants were in the Col-0 background. Mutant *ein-2-5* [65] and the transgenic line *35S:ERF1.14* [66] was provided by Antonio Molina (E.T.S.I. Agronomos, Madrid, Spain. The *sid2-1* mutant [67], Arabidopsis ecotype Kashmir 1 (Kas-1) [63] and Arabidopsis lines expressing GFP in the plasma membrane (line 29-1 encoding LTI6b) [68] were obtained from the Arabidopsis Biological Resource Center (ABRC, Ohio State University, USA). T-DNA insertional lines used in this work were: SALK_066755 (*atl9-1*), SALK_036065 (*atl9-2*), and SALK_036066 (*atl9-3*). All T-DNA insertional lines were generated by SIGnAL at the SALK Institute [69], [70]. The following gene-specific primers were used to screen for the T-DNA insertion in *ATL9*: (1) SALK_066755: 5′- TTGGCATGTAGAAACATAATTAGCG -3′(forward) and 5′-GACGACGACGACAGCACTGAA-3′ (reverse), (2) SALK_036065: 5′ –CGTTCATCTGGTCGGAGCCGTTCG-3′ (forward) and 5′-GCGTTTGATGCCTCCTTGTTG-3′ (reverse), and (3) SALK_036066: 5′ CGTTCATCTGGTCGGAGCCGTTCG-3′ (forward) and 5′-GCGTTTGATGCCTCCTTGTTG-3′ (reverse). Nested primers for confirming the T-DNA insertions in *ATL9* were as follows: (1) SALK_066755: 5′-TTGGCATGTAGAAACATAATTAGC-3′ (forward) and 5′-GACGACGACGACAGCACTGAA-3′ (reverse); (2) SALK_036065: 5′-CGATGTCGGAAGATTCTTCGG-3′ (forward) and 5′-GTCTGGCTCTCAGAACACTCC-3′ (reverse) and (3) SALK_036066: 5′- CGATGTCGGAAGATTCTTCGG-3′ (forward) and the reverse primer was the same as in SALK_036065 (Oligo 6.0 Primer Analysis Software, LSR, Minnesota, MN, USA). Seeds of homozygous lines from the three *atl9* mutants will be made available via the Arabidopsis Biological Resource Center (Ohio State University, USA). The fungal pathogens used in this work were *Golovinomyces cichoracearum* UCSC1 (Powdery mildew  =  *Erysiphe cichoracearum* UCSC1) [71]. Powdery mildew fungus was maintained as described previously [63].

### Disease Assessments

Powdery mildew inoculations and disease assessments were carried out as described in [37]. In brief, Arabidopsis seeds were planted in soil (Promix HP, Hummert International, St. Louis MO), placed in a cold room for three days and then transferred to a growth chamber (24°C day, 22°C night; 14 h day, 150 µE m^−2^ sec^−1^ of light; 60% relative humidity). After four weeks, plants were inoculated with powdery mildew and placed in a chamber under the same temperature and light conditions except at 80% relative humidity.

Disease development was assessed in a qualitative manner by monitoring the appearance of powdery symptoms on inoculated leaves over a period of 10 days post inoculation [71]. At least 36 plants per genotype were inoculated in each experiment. For quantitative assessment, inoculated leaves were treated with ethanol 100% for two hours at 65°C and 90% relative humidity before staining with trypan blue solution (25 µg/ml trypan blue in a 1∶1∶1 solution of glycerol, lactic acid and water,) for 15 minutes. Leaves were harvested for staining 6 or 7 days after inoculation depending on the development of the fungal infection. The number of conidiophores per colony was then determined for at least 36 leaves per genotype. For diaminobenzidine (DAB)/trypan blue double staining, inoculated leaves were treated with 1mg/ml DAB for two minutes under vacuum and then covered with aluminum foil for six hours at room temperature. Leaves were then immersed in 100% ethanol for two hours at 65°C and 90% of humidity before performing the trypan blue staining as described.

### Chemical Treatments

Seeds were surface sterilized and grown in liquid Murashige Skoog culture medium at a density of approximately 500 seeds (10 mg) per 125 ml flask. Flasks with seeds were incubated at 4°C for 6 days and then placed in a shaking incubator at 150 rpm for two weeks under constant illumination (125 µmol m^−2^ s^−1^) at 23°C. After fourteen days, seedlings were treated with either 100 µg ml^−1^ hydrolyzed crab shell chitin (Sigma, St Louis, MO, USA) [29] or with salicylic acid (0.5mM; Sigma, St Louis, MO, USA), jasmonic acid (5 µM; Sigma, St Louis, MO, USA) or ACC 5 µg ml^−1^ (50 µM; Sigma, St Louis, MO, USA) for thirty minutes. After 30 minutes of treatment seedlings were harvested, flash-frozen in liquid N_2_ and stored at −80°C until analysis.

### Generation of Transgenic Plants and Constructs

The *ATL9* sequence was PCR amplified using Platinum® Taq DNA Polymerase High Fidelity (Invitrogen, Carlsbad, CA). For Gateway® cloning, attB PCR primers were designed per manufacturer's recommendations (Invitrogen, Calsbad, CA). Primer sequences were as follows: attB1 forward primer 5′-GGGGACAAGTTTGTACAAAAAAGCAGGCTTC-3′; attB2 reverse primer 5′-GGGGACCAACTTTGTACAAGAAAGCTGGGTC-3′. Gene specific primers for *ATL9* were: forward primer 5′- CATACGTCGATTGGATTTTAATGG-3′ and reverse primer 5′- CCACTCGTTCATCTGGTCG-3′. Final primers used for PCR amplification of the ATL9 gene were forward 5′-GGGGACAAGTTTGTACAAAAAAGCAGGCTTCCATACGTCGATTGGATTTTAATGG-3′ and reverse 5′-GGGGACCAACTTTGTACAAGAAAGCTGGGTCCACTCGTTCATCTGGTCG-3′ using Col-0 wild type cDNA as the template. cDNA was prepared using an Amersham First-Strand cDNA Synthesis Kit (Amersham, Buckinghamshire, UK). Resulting PCR products were purified and entry clones were generated by recombination into the vector, pDONR™207 using the BP Recombination Reaction (Invitrogen, Carlsbad, CA). For the *ATL9p*:*ATL9*:GFP construct the destination vector pGWB4 (*attR1-CmR-ccdB-attR2-sGFP*) was used. The generation of the constructs for the tobacco transformation were as follows: 35S:GFP:*ATL9* utilized the destination vector pGWB6 (*35S promoter- NsGFP-attR1-CmR-ccdB-attR2*) and the destination vector pGWB5 (*35S promoter-attR-CmR-ccdB-attR2-sGFP*) was used to generate a 35S:GFP construct [68] for use as a control. All Gateway® binary vectors were kindly provided by Tsuyoshi Nakagawa at the Research Institute of Molecular Genetics, Shimane University, Japan. All plasmid inserts were sequenced prior to transformation and verified constructs were transformed into *Agrobacterium tumefaciens* strain C58C1 (pGV2260) via electroporation. Both Col-0 plants and the *atl9-1* mutant were transformed using the floral dip method [72], [73]. Kanamycin resistant plants were then selected on plates and resistant progeny were transferred to soil and allowed to set seed. The T2 progeny from these transformants were used in the experiments described in the text. The transcript levels of *ATL9* in all lines were verified using qRT-PCR as described below. Seeds of all transgenic lines will be made available via the Arabidopsis Biological Resource Center (ABRC, Ohio State University).

### Generation of Constructs for Microprojectile Bombardment

*ATL9* full length genomic DNA was amplified by attB primers containing *ATL9* sequence. Forward Primer 5′–GGGGACAAGTTTGTACAAAAAAGCAGGCTTCATGGCGATCCTCGACACAAAG–3′ and Reverse Primer 5′– GGGGACCACTTTGTACAAGAAAGCTGGGTCCACTCGTTCATCTGGTCGGAGC –3′. The resultant PCR product was cloned into pDONR221 (Invitrogen) using BP Clonase II enzyme mix (Invitrogen) to generate entry clones. The entry clones carrying *ATL9* were subcloned into T-DNA binary vector pMDC83 [74] using LR Clonase enzyme mix (Invitrogen). The resultant 35S:*ATL9*:GFP plasmid was transformed into One-Shot TOP10 Chemically Competent *E.coli* (Invitrogen) and screened on LB agar plates containing kanamycin (50 µg/mL) and hygromycin (50 µg/mL). The plasmid was isolated from positive clones using QIAprep Spin miniprep kit (Qiagen). Plasmid DNA was concentrated to 1 µg/µL by ethanol precipitation for microparticle bombardment into onion epidermal cells. The plasmid for the ER localization, ER-rk, was acquired from the ABRC (stock number CD3-959).

### Real Time Quantitative RT-PCR and Other PCR assays

Total RNA was isolated from frozen tissues using TRizol Reagent (Invitrogen®, Carlsbad, CA) according to the manufacturer's protocol. RNA samples were treated with RQ1 DNase (Promega, Madison, WI). Trace amounts of genomic DNA were removed by digestion with Turbo DNA-*free*™ (Ambion, Austin, TX). First-strand cDNA synthesis was primed with an oligo (dT)_15_ anchor primer and cDNA was synthesized using the First-Strand Synthesis Kit (Amersham-Pharmacia, Rainham, UK) according to the manufacturer's protocol. An aliquot of 1.5 µl of the first-strand synthesis reaction was used as template for PCR amplification. To ensure that the sequence amplified was specific, a nested PCR was performed using 1 µl of a 1∶50 dilution of the products synthesized in the first PCR reaction as a template. The RT-PCR, PCR and nested PCR program consisted of: 3 min at 96°C, 40 cycles of 30 s at 94°C, 30 s at 65°C, and 1 min at 72°C. The final extension step consisted of 7 min at 72°C. Amplified PCR fragments were visualized using 1.5% agarose gels.

Quantitative RT-PCR experiments were performed using a SYBR® Green qPCR kit (Finnzymes, Espoo, Finland) with reactions at a final volume of 20 µl per well and using the cycle protocol recommended by the manufacturer. Samples were run in a DNA Engine Opticon® 2 System instrument with PTC-200 DNA Engine Cycler and CFD-3220 Opticon™ 2 Detector (BioRad, Hercules, CA). Gene-specific primers were designed using the Primer Express 2.0 program (Applied Biosystems, Foster City, CA) and minimal self-hybridization and dimer formation of primers was determined using the Oligo 6.0 program (Molecular Biology Insights, West Cascade, CO). Primers with annealing temperatures of 62°C to 65°C that amplified products with lengths of about 300 bp were selected and then verified for specificity by BLAST searches. The efficiency of amplification for each gene was calculated as recommended by the manufacturer (BioRad, Hercules, CA) [75]. The following gene specific primers were used for RT-PCR and quantitative RT-PCR; *ATL9* (At2g35000): 5′-GTCGGAAGATTCTTCGGCGCATCTCC-3′ (forward) and 5′-CGACCGGACATTCGTTAATTCAAC-3′ (reverse); *PR1* (At2g14610): 5′-GATAGCCCACAAGATTATCGG-3′ (forward) and 5′-CTCGTTCACATAATTCCCACG-3′ (reverse); *ATRBOHD* (At5g47910): 5′-ATGAAAATGAGACGAGGCAATTC-3′ (forward) and 5′-GGATACTGATCATAGGCGTGGCTCC-3′ (reverse); *ATRBOHF* (At1g64060): 5′-CTTCCGATATCCTTCAACAACTC-3′ (forward) and 5′-GAGATTGCCTTTATACTATAAGTG-3′ (reverse); *MAPK3* (At3g45640): 5′-ATGAACACCGGCGGTGGCC-3′ (forward) and 5′-GGCATTCACGGGGCTGCTG-3′ (reverse); β-*ACTIN (*At3g18780): 5′-GTTGGTGATGAAGCACAATCCAAG-3′ (forward) and 5′-CTGGAACAAGACTTCTGGGCATCT-3′ (reverse). Data acquisition was performed using the Opticon Monitor Analysis software (version 2.01) and changes in transcript levels were determined by the 2^−ΔΔC^_T_ method in Microsoft EXCEL [76]. Data points were compared using a T-test. Three independent biological replicates were used in each experiment.

### Transient Expression in Tobacco and Immunoblot Analysis

Agrobacterium-mediated transient expression in tobacco was performed as described previously [77], with the exception that *Agrobacterium tumefaciens* GV3101 (at O.D._600_ of 2) was used. *Agrobacterium* containing the transformation constructs described above, 35S*:ATL9:*GFP and 35S:GFP (negative control) [68] were introduced into tobacco leaves by infiltration. After two days, infiltrated tissues were harvested for western blot analysis using the monoclonal anti-GFP antibody, JL-8 (BD Biosciences, Palo Alto, USA) at a 1∶1000 dilution [78]. Experiments were performed three times with three replicates in each experiment.

### Microparticle Bombardment of Onion Epidermal Cells

Tungsten microcarriers (1.1 µm) were prepared and coated with an equal molar ratio of 35S:*ATL9*:GFP to ER luminal marker, ER-rk [40] plasmid DNA (1 µg/µL) according to manufacturer's instructions (Bio-Rad). Fresh onion epidermal peels were transferred to MS medium with vitamins (0.5% MS Salts with vitamins, 0.7% Agar, pH 5.7) less than one hour before bombardment. Microcarriers were bombarded into onion epidermal cells using Bio-Rad PDS-1000/He Particle Delivery System (Bio-Rad). Bombardment was executed following the manufacturer's protocol under the following conditions: 900 PSI rupture disk, helium vacuum of 27 in. Hg, and a distance of 6 cm from the microcarriers to the sample. Immediately after bombardment, onion epidermal cells were incubated in the dark at room temperature (22–23°C) for 12–24 hours before observation using fluorescent microscopy.

### Microscopy and Photography Techniques

Confocal fluorescence images of Arabidopsis and tobacco tissues expressing GFP fusion constructs were observed using a Nikon Diaphot 200 inverted fluorescence microscope equipped with Nikon 60X 1.2 numerical aperture water immersion objective (Nikon, Japan) and a BioRad MRC 1024 confocal head with inverted fluorescence microscope (BioRad, Philadelphia, USA). Samples were prepared in water as previously described [68]. Confocal images were processed using Image J (v. 1.30, N.I.H. USA), Cas40 (Confocal Assistant v. 4.02, USA) and Adobe Photoshop 7.0 (Adobe Systems Inc., San Jose, CA) programs.

For subcellular localization in onion cells using fluorescent microscopy, onion epidermal cells were mounted in distilled H_2_O ∼12 hours after bombardment. GFP and mCherry fluorophores were visualized concurrently with a Nikon Eclipse 90i epi-fluorescent microscope (Nikon, Melville, NY) equipped with an OptiGrid imaging system (Qioptiq, Paris, France) using the FITC HYQ (Excitation: 460–500 nm; Emission: 510–560 nm) and TRITC HYQ (Excitation: 530–560 nm; Emssion: 590–650 nm) filters. Images were generated and/or merged using NIS-Elements software (Version 3.2, Nikon).

For light microscopy experiments, a Nikon Eclipse E600 light microscope was used and images were recorded with a Nikon Spot Advance 32 camera (Nikon, Japan). To determine the number of conidiophores per fungal colony, leaves were inoculated at low density with *G. cichoracearum* and stained with trypan blue either 6 or 7 dpi [37], [71]. To assess cell death, leaves were stained with trypan blue in phenol [79]. Photographs of Arabidopsis leaves inoculated with *G. cichoracearum* were taken using a Nikon Coolpix E995. Tissues that were double stained with Trypan blue (TB) and Diamino-benzidine (DAB) were first stained with DAB and subsequently with TB. Osmotic shock was induced by immersion of Arabidopsis leaves in sodium chloride 0.5 M for 10 minutes.

### Ubiquitination Assays and Cloning of ATL9 for expression assays

Total RNA isolated from leaves of 2–4 week old *Arabidopsis thaliana* ecotype Col-0 plants was used in reverse transcription reactions followed by PCR to amplify the predicted open reading frame (ORF) of *ATL9* (At2g35000). The Qiagen RNeasy plant RNA extraction kit (Qiagen, Valencia, CA) was used to isolate total RNA as per manufacturer instructions. The amplified cDNA was first introduced into the Gateway entry vector, pDONR (Invitrogen) and the DNA sequence analyzed. ORF's determined to be correct were introduced into the pDEST15 (Invitrogen, Carlsbad, CA) protein expression vector to produce in-frame fusions with the GST tag. *At*UBC8 was cloned in a similar manner and introduced into the pDEST17 (Invitrogen, Carlsbad, CA) vector to produce an in-frame fusion with the 6X HIS tag. The expression clone containing yeast E1 was provided by M. Wogulis (University of California-Davis). Ubiquitination assays were carried out as described previously [80]. Reactions (30 mL) containing 50 mM Tris-HCl, pH 7.5; 10 mMMgCl2; 0.05 mM ZnCl2; 1 mM ATP; 0.2 mM dithiothreitol; 10 mM phosphocreatine;0.1 unit of creatine kinase (Sigma); 50 ng of yeast E1 (Boston Biochem, Cambridge, MA); 250 ng of purified E2 AtUBC8; 250 ng of eluted/bead-bound GST-RING, GST-mutated RING protein, or GST-CIP8 (positive control); and 2 mg ubiquitin (Sigma) were incubated at 30°C for 2 h. Reactions were stopped by adding 6 mL of 53 SDS-PAGE sample buffer (125 mM Tris-HCl, pH 6.8, 20% [v/v] glycerin, 4% [w/v] SDS, and 10% [v/v] β-mercaptoethanol) and analyzed by SDS-PAGE electrophoresis followed by western blotting using ubiquitin antibodies. For zinc-chelating experiments, bead-bound GST-RING protein was either incubated in 50 mM Tris-HCl, pH 7.4, containing 5 mM TPEN or 0.5% (v/v) ethanol (vehicle; mock treatment) for 16 h at 4°C with at least three solution changes. Beads were then washed three times in 50 mM Tris-HCl, pH 7.4. An aliquot of TPEN-treated bead-bound GST-RING protein was incubated with 1 mM ZnCl_2_ for 4 h at 4°C with at least three solution changes, followed by three washes in 50 mM Tris-HCl, pH 7.4. Mock-, TPEN-, and TPEN plus ZnCl_2_-treated bead-bound GST-RING protein were then used in ubiquitination assays.

### Protein expression and purification

GST:*ATL9* fusions were expressed in *E. coli* strain BL21 (DE3) pLysS in 50 ml cultures. Transformed cells were grown at 37°C for 2 to 3 hours or to an OD600 of 0.4–0.6 before induction with 0.5 mM IPTG for 3 to 4 hours at 37°C. Cells were harvested by centrifugation and lysed in 2 ml of lysis buffer containing 25 mM Tris-HCl pH 7.5, 500 mM NaCl, and 0.01% Triton X-100. For purification, 100 µl of glutathione sepharose beads (Sigma-Aldrich, St. Louis, MO) was added to cleared lysates and incubated for two hours at 4°C. Beads were then washed four times with 1 ml of wash buffer containing 25 mM Tris-HCl pH 7.5, 300 mM NaCl, and 0.01% (v/v) Triton X-100. GST fusion proteins were eluted with 100 µl of elution buffer containing 25 mM Tris-HCl pH 7.5, 150 mM NaCl, and 0.01% (v/v) Triton X-100 supplemented with 15 mM reduced glutathione or the GST fusion proteins were left bound to the beads. 40 µl of glycerol was added to the eluted protein. Bead bound GST proteins were stored in 25 mM Tris-HCl pH 7.5, 150 mM NaCl, 0.01% (v/v) Triton X-100 and 40% (v/v) glycerol. Proteins were stored at −80°C. 6X HIS tagged *At*UBC8 was expressed and purified in a similar manner. The 6X HIS fusion was expressed in *E. coli* strain BL21 AI and induced with 0.2% (w/v) arabinose. Lysis and wash buffer were supplemented with 5 mM imidazole and elution buffer supplemented with 300 mM imidazole. Yeast E1 was expressed as an Intein fusion and purified using the IMPACT (Intein mediated purification with an affinity chitin-binding tag) system as per the manufacturer's instructions (New England Biolabs, Beverly, Massachusetts, USA). SDS-page electrophoresis followed by Coomassie blue staining and Bradford assays (Biorad, Philadelphia, USA) were used to quantify purified proteins. Western blot analysis using GST antibodies or HIS antibodies (Amersham, Buckinghamshire, UK) was also used to confirm the presence and integrity of the fusion protein.

### Bioinformatics Analysis

Additional data analysis and information about gene expression of *ATL9* was obtained from the following web pages: http://affymetrix.arabidopsis.info/narrays/, https://www.genevestigator.ethz.ch/. Web analysis was used to obtain microarray data regarding *ATL9* gene expression in different tissues, conditions, and treatments and to examine co-expression patterns of *ATL9* and other genes. The Aramemnon program (http://aramemnon.botanik.uni-koeln.de/) was used to analyze the structure of *ATL9* and to predict its cellular localization. Tools used for general biomolecular analysis, BLAST, sequence alignment and determination of protein-specific domains were: http://www.us.expasy.com/tools/, http://www.ncbi.nlm.nih.gov/, and http://www.ebi.ac.uk/Tools/.

### Microarray Expression Analysis

Seedlings of *ATL9-1* and Col-0 were treated with hydrolyzed crab shell chitin or with water as a mock control in liquid culture for 30 min as described previously [29]. Four independent biological replicates were performed for each experiment. RNA extractions, labeling and hybridization to ATH1 Affymetrix GeneChips® (Affymetrix GeneChip Expression Analysis Technical Manual, Affymetrix, Inc., Santa Clara, CA) were performed as described previously [81] with the exception that 15 µg of RNA were biotinylated to obtain cRNA. Data were extracted from the GeneChip® images using MAS5.0 (Affymetrix) software and imported into GeneSpring 6.0 software (Silicon Genetics, Redwood City, CA) for normalization and further analysis. Fold change threshold is shown in Table1. This data set has been made available via the Gene Expression Omnibus (GSE2169) (http://www.ncbi.nlm.nih.gov/geo/).

## Supporting Information

Figure S1 Transient Expression of ATL9 in Tobacco Epidermal Cells. A) Expression of 35S:GFP:ATL9 construct in tobacco epidermal cells shows localization to the ER with no nuclear localization. B) Expression of 35S:GFP negative control in tobacco epidermal cells shows GFP localizing to the nucleus when it is not fused to ATL9. C) Western blot confirming sizes of ATL9 and GFP fusion proteins from A and B. Blot was probed with a monoclonal anti-GFP antibody. Bars: 5 µm.
(1.42 MB TIF)

Table S1 Microarray data for selected chitin-responsive genes.
(0.18 MB DOC)
