# Supplementary figures and images for: ATL9, a RING Zinc Finger Protein with E3 Ubiquitin Ligase Activity Implicated in Chitin- and NADPH Oxidase-Mediated Defense Responses
Source: PLoS One. 2010 Dec 23;5(12):e14426. doi: 10.1371/journal.pone.0014426 (PMC3009710; doi:10.1371/journal.pone.0014426)

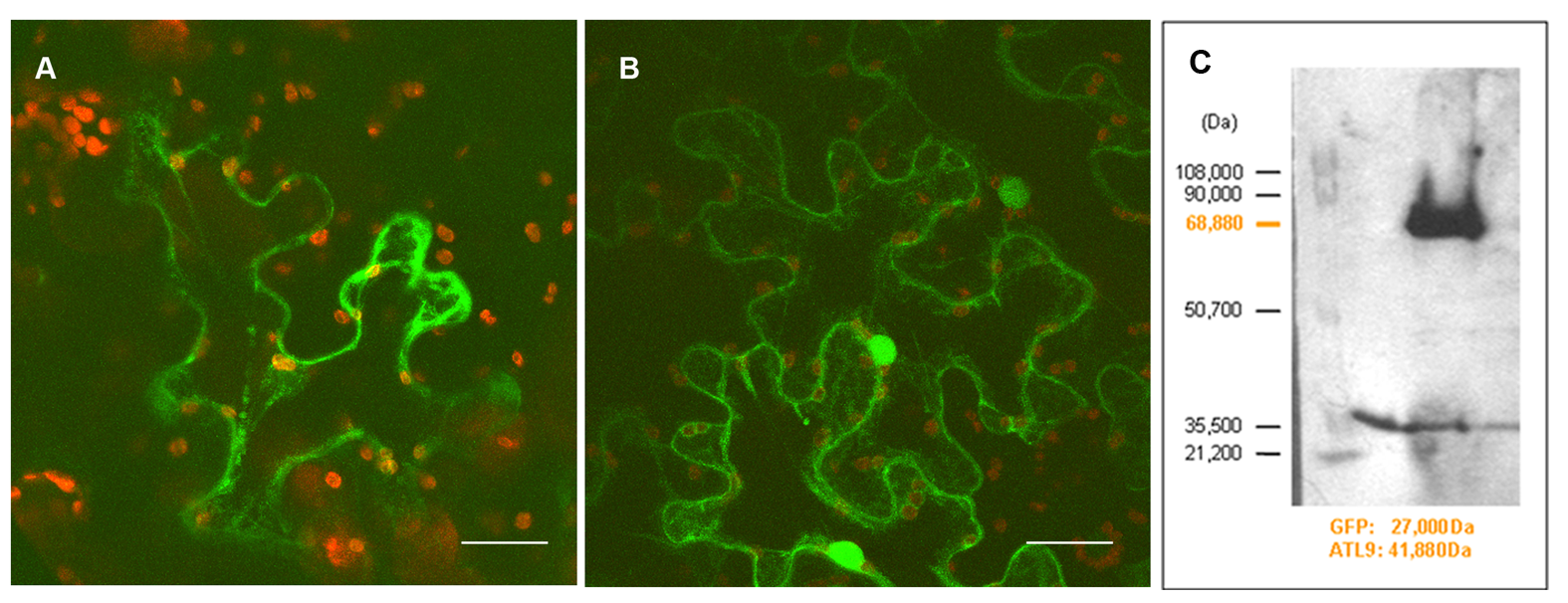

Supplement: Figure S1 — Transient Expression of ATL9 in Tobacco Epidermal Cells. A) Expression of 35S:GFP:ATL9 construct in tobacco epidermal cells shows localization to the ER with no nuclear localization. B) Expression of 35S:GFP negative control in tobacco epidermal cells shows GFP localizing to the nucleus when it is not fused to ATL9. C) Western blot confirming sizes of ATL9 and GFP fusion proteins from A and B. Blot was probed with a monoclonal anti-GFP antibody. Bars: 5 µm. (1.42 MB TIF) [file pone.0014426.s001.tif]
