# Supplementary material for: ATL9, a RING Zinc Finger Protein with E3 Ubiquitin Ligase Activity Implicated in Chitin- and NADPH Oxidase-Mediated Defense Responses
Source: PLoS One. 2010 Dec 23;5(12):e14426. doi: 10.1371/journal.pone.0014426 (PMC3009710; doi:10.1371/journal.pone.0014426)
Supplement: Table S1 — Microarray data for selected chitin-responsive genes. (0.18 MB DOC) [file pone.0014426.s002.doc]

| ***Expression levels:*** | | ***Mock plants (normalized)*** | | ***Treated plants (normalized)*** | |
| --- | --- | --- | --- | --- | --- |
| **Gene Identification and/or Putative Function** | **AGI Code** | **Wild Type** | ***atl9-A*** | **Wild Type** | ***atl9-A*** |
| **Selected Defense Related Genes** | | | | | |
| ATL2- ACRE132 | At3g16720 | 1.075 | 0.984 | 4.510 | 5.186 |
| ATL6 | At3g05200 | 1.012 | 0.904 | 2.127 | 2.748 |
| ATL17 | At4g15975 | 1.088 | 1.570 | 6.060 | 9.011 |
| ACRE1b | At4g17490 | 1.012 | 0.995 | 9.047 | 11.277 |
| BAK1 | At4g33430 | 1.018 | 1.018 | 1.432 | 1.472 |
| FLS2 | At5g46330 | 1.000 | 1.230 | 1.605 | 1.180 |
| LysM RLK1 | At3g21630 | 1.007 | 1.021 | 1.574 | 1.810 |
| EFR-EF-Tu | At5g20480 | 0.981 | 1.047 | 1.382 | 1.426 |
| PP2C | At5g59220 | 1.114 | 1.366 | 0.484 | 1.111 |
| ABI5 | At1g59260 | 1.006 | 1.238 | 0.552 | 0.689 |
| PP2C- ABI1 | At4g26080 | 1.010 | 1.151 | 0.760 | 0.829 |
| PP2C- ABI2 | At5g57050 | 0.997 | 1.239 | 0.508 | 0.491 |
| NCED3 (ABA synthesis) | At3g14440 | 0.887 | 0.856 | 0.675 | 0.851 |
| CESA8/IRX1/ERN1 | At4g18780 | 0.822 | 1.222 | 1.118 | 1.030 |
| CESA4/IRX5/NWS2 | At5g44030 | 0.932 | 1.058 | 0.996 | 0.996 |
| Lox-3, lipoxygenase | At1g17420 | 0.859 | 1.085 | 6.156 | 5.490 |
| MAP kinase 3 | At3g45640 | 1.005 | 0.947 | 2.222 | 2.580 |
| MAP kinase kinase 4 (ATMKK4) | At1g51660 | 0.983 | 1.011 | 2.214 | 2.415 |
| MAP kinase kinase 5 | At3g21220 | 1.017 | 1.490 | 1.910 | 2.126 |
| NPR1-1 | At4g2612 | 1.002 | 0.988 | 2.221 | 2.174 |
| DRP-like | At1g66090 | 1.000 | 1.380 | 9.257 | 13.274 |
| DRP-like | At1g72950 | 1.026 | 1.643 | 9.655 | 17.739 |
| DRP-like | At1g72920 | 1.086 | 1.764 | 3.626 | 5.133 |
| EDS1 | At3g48090 | 1.003 | 0.981 | 2.281 | 1.923 |
| RAR1 | At5g51700 | 1.005 | 1.099 | 0.932 | 1.122 |
| SGT1 | At4g08320 | 1.059 | 1.016 | 1.014 | 0.985 |
| HSP90 | At3g07770 | 1.009 | 1.015 | 0.921 | 0.934 |
| Coi1 | At2g39940 | 0.981 | 1.020 | 0.912 | 0.924 |
| ETR1 | At1g66340 | 1.003 | 0.853 | 1.064 | 0.870 |
| EDS5 | At4g39030 | 1.002 | 1.059 | 1.535 | 1.554 |
| EIN3 | At3g20770 | 0.997 | 0.942 | 0.973 | 1.070 |
| PAD2 | At5g66140 | 1.008 | 0.938 | 1.125 | 0.857 |
| PAD4 | At3g52430 | 0.966 | 1.094 | 3.531 | 4.194 |
| PDF1.2 | At5g44420 | 0.946 | 1.073 | 0.384 | 1.220 |
| JAR1 | At2g46370 | 0.969 | 1.086 | 1.331 | 1.080 |
| AtrbohD | At5g47910 | 0.997 | 0.979 | 1.695 | 2.201 |
| AtrbohF | At1g64060 | 0.953 | 0.938 | 0.762 | 0.970 |
| AtrbohC | At5g51060 | 0.962 | 1.022 | 1.169 | 0.916 |
| AtPEN1 | At4g15340 | 0.996 | 1.374 | 0.382 | 1.077 |
| AtPEN2 | At2g44490 | 1.000 | 1.003 | 1.927 | 1.962 |
| AtPEN3 | At5g36150 | 1.100 | 0.515 | 2.132 | 0.693 |
| SAG101 | At5g14930 | 0.977 | 1.000 | 1.972 | 1.888 |
| **Selected Genotype specific genes** |  |  |  |  |  |
| SigA binding protein | At2g41180 | 1.027 | 0.097 | 0.920 | 0.438 |
| ICS1, sid2 (isochorismate synthase,) | At1g74710 | 1.032 | 0.768 | 2.494 | 1.230 |
| PCC1 (pathogen and circadian controled1) | At3g22231 | 0.997 | 0.707 | 2.391 | 0.579 |
| Peroxidase | At5g64120 | 1.061 | 1.107 | 3.099 | 1.519 |
| SERINE CARBOXYPEPTIDASE II-like protein | At4g30610 | 0.988 | 0.702 | 1.633 | 0.667 |
| pathogenesis related-1 (PR1) protein | At2g14580 | 0.967 | 0.361 | 0.892 | 0.330 |
| helix-loop-helix DNA-binding protein | At1g73830 | 1.023 | 0.398 | 1.365 | 0.585 |
| cytochrome p450 family | At4g12330 | 0.950 | 2.336 | 0.358 | 1.280 |
| Cupin, germin-like protein | At3g05950 | 0.902 | 1.410 | 0.158 | 0.990 |
| hydroxynitrile lyase like protein | At4g15100 | 1.337 | 2.019 | 0.061 | 2.464 |
| flavonol sulfotransferase | At1g74090 | 0.999 | 2.108 | 0.659 | 1.584 |
| pentacyclic triterpene synthase | At5g42600 | 0.639 | 1.515 | 0.063 | 1.465 |
| myrosinase-associated protein, | At1g54020 | 1.000 | 2.390 | 0.290 | 2.574 |
| stearoyl-acyl carrier protein desaturase | At3g02610 | 0.812 | 2.141 | 0.292 | 1.612 |
| tyrosine aminotransferase (AtTAT1) | At4g23600 | 0.927 | 2.499 | 0.339 | 2.212 |
| glycosyl hydrolase family 14 (beta-amylase) | At4g15210 | 0.802 | 2.326 | 0.123 | 1.899 |
| receptor-like kinase, sugar binding | At1g61610 | 1.097 | 1.892 | 3.372 | 2.875 |
| peroxidase | At5g64120 | 1.061 | 1.107 | 3.099 | 1.519 |
| peroxidase | At5g64100 | 0.936 | 0.930 | 2.115 | 1.030 |
| Gamma-thionin plant defensin protein, (PDF2.5) | At5g63660 | 0.880 | 0.670 | 1.641 | 0.644 |
| proline-rich cell wall protein family | At2g22510 | 0.885 | 0.771 | 1.682 | 0.855 |
| arabinogalactan-protein (AGP9) | At2g14890 | 0.987 | 0.972 | 1.812 | 0.998 |
| expansin | At1g20190 | 1.031 | 0.966 | 3.042 | 0.792 |
| ammonium transporter | At1g64780 | 0.950 | 1.045 | 1.448 | 0.635 |
| transcription factor | At1g44830 | 0.973 | 0.902 | 3.680 | 1.703 |
| pectinesterase (pectin methylesterase) | At4g22010 | 1.053 | 1.161 | 2.868 | 0.998 |
| F-box, late embryogenesis abundant | At1g61340 | 1.070 | 1.237 | 9.051 | 18.137 |
| Expressed protein | At2g31945 | 1.031 | 1.178 | 0.442 | 3.799 |
| WAK-cell wall associated kinase | At1g79680 | 0.980 | 1.016 | 3.507 | 6.070 |
| lysine-ketoglutarate reductase/saccharopine | At4g33150 | 1.016 | 1.191 | 0.476 | 1.337 |
| 2OG-Fe(ll) dioxygenase | At1g14120 | 1.039 | 1.651 | 0.583 | 1.695 |
| UDP-glucose transferase(UGT1) | At1g05560 | 0.970 | 1.140 | 0.438 | 1.218 |
| LRR leucine rich repeat protein | At5g12940 | 1.145 | 0.949 | 4.445 | 1.608 |
| **Selected Genotype and Treatment specific genes** | | | | | |
| cytochrome p450 family | At2g30770 | 1.084 | 0.611 | 1.620 | 0.674 |
| flavonol 3-O-glucosyltransferase | At5g54060 | 0.817 | 0.972 | 0.678 | 1.516 |
| PMEI;pectin methylesterase inhibitor | At1g70720 | 0.963 | 3.076 | 0.877 | 3.671 |
| hypothetical protein (unknown) | At2g03540 | 0.934 | 5.134 | 7.456 | 11.040 |
| hypothetical protein (unknown) | At2g36440 | 1.291 | 10.948 | 271.151 | 403.382 |
| hypothetical protein (unknown) | At2g03540 | 0.934 | 5.134 | 2.039 | 7.456 |
| protein, (cell cycle related) | At3g43250 | 0.895 | 3.399 | 6.574 | 13.502 |
| GAST1-like protein | At1g74670 | 1.012 | 0.735 | 2.138 | 1.086 |
| CARBONIC ANHYDRASE 2 | At5g14740 | 1.043 | 0.628 | 1.945 | 1.196 |
| light-harvesting chlorophyll a/b binding protein | At5g54270 | 0.981 | 1.306 | 2.002 | 1.335 |
| light-harvesting chlorophyll a/b binding protein | At3g54890 | 0.991 | 1.221 | 1.996 | 1.227 |
| photosystem I subunit V precursor | At1g55670 | 0.979 | 1.160 | 1.848 | 1.090 |
| glycosyl hydrolase family 35 (beta-galactosidase) | At2g28470 | 0.974 | 1.035 | 1.905 | 1.007 |
| glycosyl hydrolase family 3 | At1g02640 | 1.094 | 0.899 | 2.476 | 1.125 |
| glycosyl hydrolase family 35 (beta-galactosidase) | At5g63810 | 0.878 | 0.748 | 1.912 | 0.794 |
| lipase/hydrolase | At1g29670 | 0.946 | 1.146 | 2.970 | 1.645 |
| putative fatty acid elongase | At2g15090 | 0.973 | 0.762 | 1.859 | 1.285 |
| protein phosphatase | At5g02760 | 0.954 | 0.571 | 1.075 | 0.260 |
| nucleoid DNA-binding protein cnd41 | At5g10760 | 1.078 | 0.915 | 3.052 | 0.747 |
| xyloglucan endotransglycosylase, | At1g11545 | 1.003 | 0.740 | 3.458 | 0.748 |
| xyloglucan endotransglycosylase (ext/EXGT-A1) | At2g06850 | 0.956 | 0.741 | 2.128 | 0.680 |
| xyloglucan endotransglycosylase, | At3g23730 | 1.009 | 0.679 | 1.846 | 0.860 |
| fasciclin-like arabinogalactan-protein (FLA9) | At1g03870 | 0.967 | 0.780 | 2.084 | 0.750 |
| arabinogalactan-protein (AGP18) | At4g37450 | 0.995 | 1.123 | 2.215 | 0.884 |
| nodulin-like protein | At4g30420 | 0.972 | 0.699 | 2.034 | 0.768 |
| pectate lyase | At5g48900 | 1.019 | 0.824 | 2.223 | 0.767 |
| pectate lyase A11 | At1g04680 | 1.015 | 1.127 | 2.254 | 1.090 |
| Serine carboxipeptidase II | At4g30610 | 0.987 | 0.702 | 1.633 | 0.667 |
| putative receptor-like protein kinase, DUF26 | At4g04500 | 1.079 | 0.705 | 2.598 | 0.756 |
| cytochrome p450 family | At4g12330 | 0.949 | 2.336 | 1.279 | 0.716 |
| FA desaturase, stearoyl-acyl carrier protein desaturase | At3g02610 | 0.812 | 2.141 | 1.612 | 0.955 |
| myrosinase-associated protein, | At1g54020 | 1.000 | 2.390 | 2.574 | 0.853 |
| glycosyl hydrolase family 14 (beta-amylase) | At4g15210 | 0.802 | 2.326 | 1.899 | 1.067 |
| hydroxynitrile lyase like protein | At4g15100 | 1.337 | 2.019 | 2.464 | 0.861 |
